# Regional rather than global brain age mediates cognitive function in cerebral small vessel disease

**DOI:** 10.1093/braincomms/fcac233

**Published:** 2022-09-14

**Authors:** Pei-Lin Lee, Chen-Yuan Kuo, Pei-Ning Wang, Liang-Kung Chen, Ching-Po Lin, Kun-Hsien Chou, Chih-Ping Chung

**Affiliations:** Institute of Neuroscience, National Yang Ming Chiao Tung University, Taipei, Taiwan; Aging and Health Research Center, National Yang Ming Chiao Tung University, Taipei, Taiwan; Aging and Health Research Center, National Yang Ming Chiao Tung University, Taipei, Taiwan; Department of Neurology, Neurological Institute, Taipei Veterans General Hospital, Taipei, Taiwan; Brain Research Center, National Yang Ming Chiao Tung University, Taipei, Taiwan; Center for Geriatric and Gerontology, Taipei Veterans General Hospital, Taipei, Taiwan; Aging and Health Research Center, National Yang Ming Chiao Tung University, Taipei, Taiwan; Center for Geriatric and Gerontology, Taipei Veterans General Hospital, Taipei, Taiwan; Taipei Municipal Gan-Dau Hospital (managed by Taipei Veterans General Hospital), Taipei, Taiwan; Institute of Neuroscience, National Yang Ming Chiao Tung University, Taipei, Taiwan; Brain Research Center, National Yang Ming Chiao Tung University, Taipei, Taiwan; Department of Biomedical Imaging and Radiological Sciences, National Yang Ming Chiao Tung University, Taipei, Taiwan; Institute of Neuroscience, National Yang Ming Chiao Tung University, Taipei, Taiwan; Brain Research Center, National Yang Ming Chiao Tung University, Taipei, Taiwan; Aging and Health Research Center, National Yang Ming Chiao Tung University, Taipei, Taiwan; Department of Neurology, Neurological Institute, Taipei Veterans General Hospital, Taipei, Taiwan

**Keywords:** cerebral small vessel disease, brain age

## Abstract

The factors and mechanisms underlying the heterogeneous cognitive outcomes of cerebral small vessel disease are largely unknown. Brain biological age can be estimated by machine learning algorithms that use large brain MRI data sets to integrate and compute neuroimaging-derived age-related features. Predicted and chronological ages difference (brain-age gap) reflects advanced or delayed brain aging in an individual. The present study firstly reports the brain aging status of cerebral small vessel disease. In addition, we investigated whether global or certain regional brain age could mediate the cognitive functions in cerebral small vessel disease. Global and regional (400 cortical, 14 subcortical and 28 cerebellum regions of interest) brain-age prediction models were constructed using grey matter features from MRI of 1482 healthy individuals (age: 18–92 years). Predicted and chronological ages differences were obtained and then applied to non-stroke, non-demented individuals, aged ≥50 years, from another community-dwelling population (I-Lan Longitudinal Aging Study cohort). Among the 734 participants from the I-Lan Longitudinal Aging Study cohort, 124 were classified into the cerebral small vessel disease group. The cerebral small vessel disease group demonstrated significantly poorer performances in global cognitive, verbal memory and executive functions than that of non-cerebral small vessel disease group. Global brain-age gap was significantly higher in the cerebral small vessel disease (3.71 ± 7.60 years) than that in non-cerebral small vessel disease (−0.43 ± 9.47 years) group (*P* = 0.003, η^2^ = 0.012). There were 82 cerebral cortical, 3 subcortical and 4 cerebellar regions showing significantly different brain-age gap between the cerebral small vessel disease and non-cerebral small vessel disease groups. Global brain-age gap failed to mediate the relationship between cerebral small vessel disease and any of the cognitive domains. In 89 regions with increased brain-age gap in the cerebral small vessel disease group, seven regional brain-age gaps were able to show significant mediation effects in cerebral small vessel disease-related cognitive impairment (we set the statistical significance *P* < 0.05 uncorrected in 89 mediation models). Of these, the left thalamus and left hippocampus brain-age gap explained poorer global cognitive performance in cerebral small vessel disease. We demonstrated the interconnections between cerebral small vessel disease and brain age. Strategic brain aging, i.e. advanced brain aging in critical regions, may be involved in the pathophysiology of cerebral small vessel disease-related cognitive impairment. Regional rather than global brain-age gap could potentially serve as a biomarker for predicting heterogeneous cognitive outcomes in patients with cerebral small vessel disease.

## Teaser

Lee *et al*. construct global and 442 regional brain-age prediction models and reveal both the global and region-specific advanced brain aging in cerebral small vessel disease. There are mediation effects of regional rather than global brain ages in cognitive impairment related to cerebral small vessel disease. Strategic brain aging may be involved in the pathophysiology of cerebral small vessel disease.

## Graphical Abstract

**Graphical Abstract:**
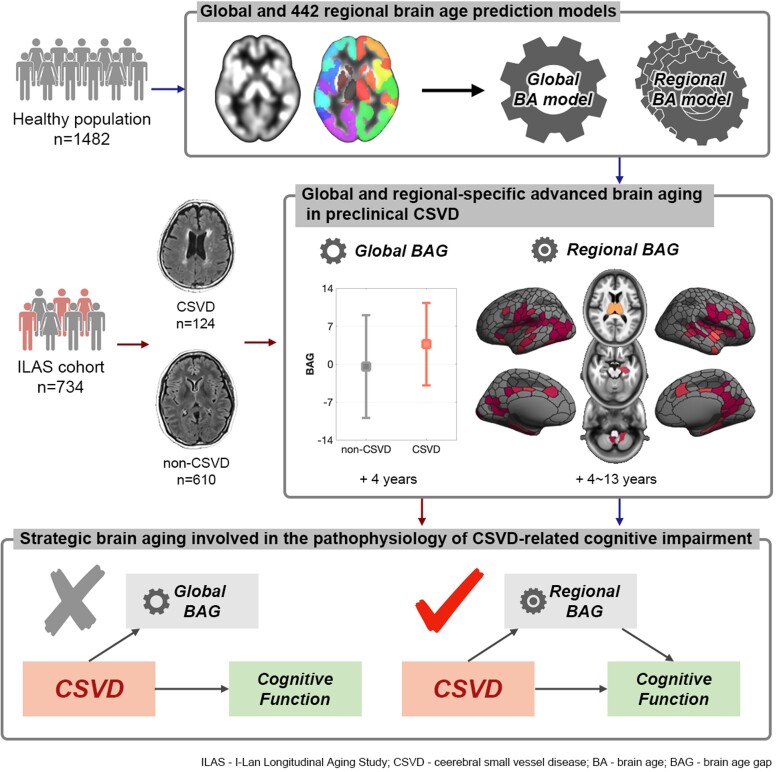

## Introduction

Human life expectancy has extended substantially in the past two centuries. As a result, age-related morbidities pose a rising challenge in many developed and developing countries.^1^ To achieve healthy aging, the processes involved in maintaining functional ability to ensure well-being in older ages, identifying who and how they deviate from healthy aging trajectories is important. An individual’s biological age may differ from chronological age and is a better indicator for one’s age-related health status.^2^ Brain biological age can be estimated by machine learning algorithms that use large brain MRI data sets to integrate and compute neuroimaging-derived age-related features.^3,4^ This neuroimaging-based brain-age measure reflects advanced or delayed brain aging in an individual, based on the difference between predicted brain age (brain biological age) and chronological age, that is, the brain-age gap (BAG) estimate. The BAG is used to evaluate how neurological diseases influence the normal brain aging process, and predict clinical outcomes in patients with these diseases.,^3,4^ Sporadic cerebral small vessel disease (CSVD) is a manifestation of age-related pathologies of the brain vasculature such as arteriosclerosis/lipohyalinosis and cerebral amyloid angiopathy.^5^ Nowadays, it can be diagnosed pre-mortem with corresponding brain parenchyma lesions noted on MRI, including white matter hyperintensities (WMHs), lacunes, and cerebral microbleeds (CMBs).^5,6^ CSVD is the most common cause of vascular cognitive impairment and dementia in the elderly and also plays an important role in stroke and neurodegenerative diseases such as Alzheimer’s disease.^5^ Although its clinical significance is well acknowledged, how CSVD exerts its influence on the brain aging process and their interactive effects on cognitive functions are largely unknown.

The present study analyzed a brain anatomical MRI data set from a community-based middle-to-old-aged non-stroke and non-demented population (≥ 50 years old). The study evaluated the pathophysiological burden of CSVD in the brain at the onset or early stage of the disease. To estimate individual brain age unbiasedly, a brain-age prediction model was established based on an independent large sample-sized brain MRI data set. In addition to the global brain age, regional brain age in 400 cortical regions of interest (ROIs), 14 subcortical ROIs and 28 cerebellum ROIs were estimated for mapping the detailed spatial distribution of the accelerated brain aging pattern in each individual with asymptomatic CSVD (covert CSVD: non-demented stroke-free). First, we investigated whether CSVD would influence the brain aging process at the asymptomatic stage by evaluating the global and regional BAG in participants with and without CSVD. We hypothesized that advanced brain aging would have already occurred at an asymptomatic early stage of the disease. Second, we determined if advanced brain aging is associated with cognitive functions in CSVD and region-specific, and whether strategic advanced brain aging is involved in the pathophysiology of CSVD-related cognitive impairment.

## Materials and methods

### Participants—the community-dwelling cohort for CSVD analysis

Participants with CSVD analysis were from the I-Lan Longitudinal Aging Study (ILAS) cohort, which is a community-based aging cohort study in I-Lan County, Taiwan, that aims to evaluate the mechanisms of aging.^7^ Community-dwelling adults, aged ≥50 years, from the Yuanshan Township in I-Lan County were invited to participate. The inclusion criteria of the ILAS were as follows: (i) inhabitants of I-Lan County who were not planning to move soon, and (ii) aged ≥50 years. Participants who met any of the following conditions were excluded: (i) inability to communicate and complete an interview, (ii) inability to complete a simple motor task (e.g. a 6 m walk) due to functional disability, (iii) presence of any major illness with associated decreased life expectancy (less than 6 months), (iv) presence of any contraindication for MRI (such as metal implants) and (v) institutionalization for any reason. Participants diagnosed with neuropsychiatric diseases, such as dementia, stroke, brain tumour or major depression, were also excluded from the present study. The present study used demographic information, cognitive assessments and multimodal brain MRI data from the initial sampling wave of the ILAS (recruited between January 2011 and July 2014).

### Participants—independent cohort data set for constructing a brain-age prediction model

To construct robust global and regional brain-age prediction models with larger sample sizes, the T_1_-weighted anatomical scans of healthy participants were drawn from multiple image data sets of our previous studies.^8-12^ All included participants were free of neuropsychiatric diseases and had no history of head trauma or other major medical conditions. A total of 1482 healthy individuals (age range = 18–92 years; males = 681, females = 801) were included in the training and validation of the constructed global and regional brain-age prediction models. Detailed information regarding the MR scanner, image acquisition protocol and demographic variables of each data set are listed in Supplementary Table 1 and Supplementary Figure 1.

### Multimodal brain MRI acquisition in the ILAS cohort

Multimodal neuroimaging acquisition was performed at the National Yang Ming Chiao Tung University in Taiwan to obtain image-based brain parenchyma CSVD markers and grey matter volume (GMV) information for each participant in the ILAS cohort. All brain MRI scans were collected on a single 3T Siemens MRI scanner (Siemens Magnetom Tim Trio, Erlangen, Germany) with an identical vendor-supplied 12-channel phased-array head coil and imaging protocols. The participants’ head position was stabilized with cushions during scans, and all MRI scans were acquired without inter-slice gap or interpolation. The imaging sequences were as follows. First, to extract grey matter (GM) volume information for each individual, sagittal T_1_-weighted anatomical scans were acquired using the three-dimensional T_1_-weighted magnetization-prepared rapid-acquisition gradient-echo sequence [repetition time (TR)/echo time (TE)/inversion time (TI) = 3500/3.5/1100 ms; flip angle = 7°; number of excitations (NEXs) = 1; field of view (FOV) = 256 × 256 mm; matrix size = 256 × 256; 192 slices; and voxel size = 1.0 mm^3^). Second, to evaluate individual lacune and WMH, axial T_2_-weighted fluid-attenuated inversion recovery (FLAIR) images were acquired using a two-dimensional T_2_-weighted FLAIR multi-shot turbo-spin-echo sequence (TR/TE/TI = 9000/143/2500 ms; flip angle = 130°; NEX = 1; FOV = 220 × 220 mm; matrix size = 320 × 320, echo train length = 35; 63 slices; and voxel size = 0.69 mm × 0.69 mm × 2.0 mm). Finally, to evaluate individual’s CMBs, axial susceptibility-weighted images (SWIs) were acquired using a three-dimensional SWI sequence (TR/TE = 28/21 ms; flip angle = 15°, FOV = 256 × 224 mm; matrix size = 256 × 224; 88 slices; bandwidth = 120 Hz/Px; and voxel size = 1.0 × 1.0 × 2.0 mm).

### Brain-age prediction model construction and applications

**Figure 1:**
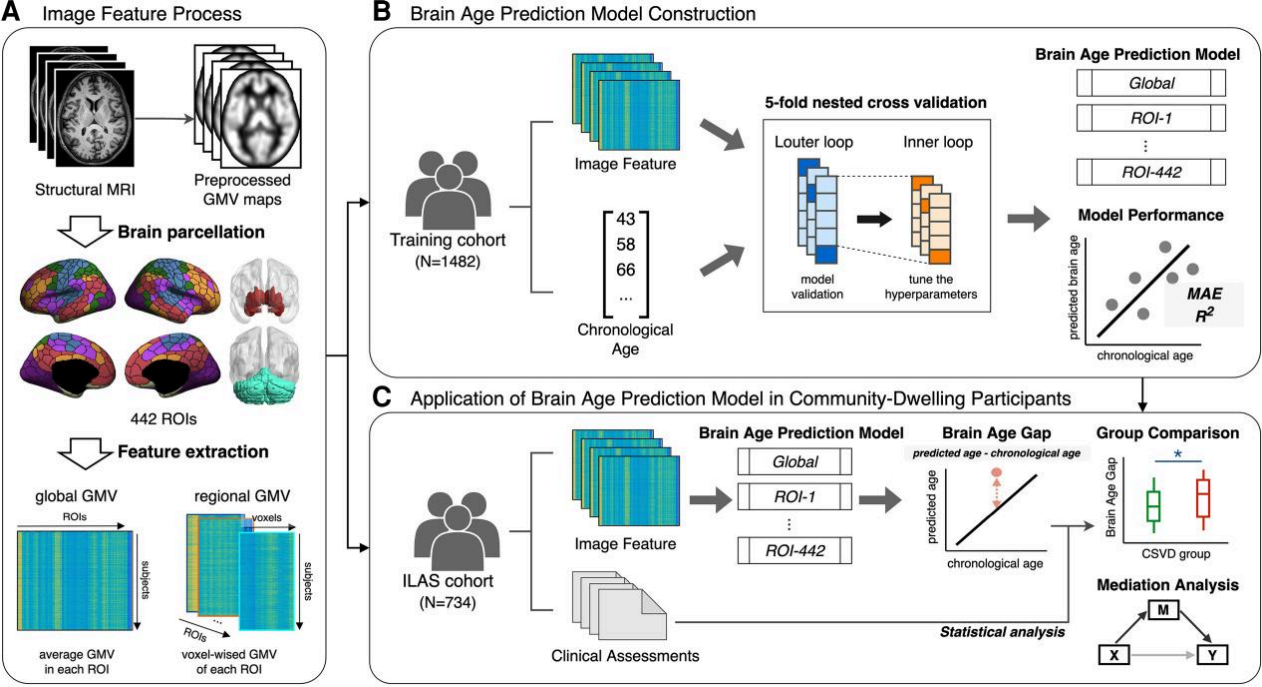
**Study design framework.** (**A**) The structural MRI data went through the VBM preprocessing pipeline and the voxel-wised GMV features were extracted. (**B**) The image features were used to construct and validate the brain-age predictive model in the training data set. The performances of models were tested and the best-performed model was selected. (**C**) The established model was applied to another community-dwelling cohort (ILAS) to estimate individual’s BAG. The statistical analysis was further conducted to compare the difference of BAG between CSVD and non-CSVD groups and the mediating role of BAG between CSVD and related cognitive impairments. CSVD = cerebral small vessel disease; GMV = grey matter volume; ILAS = I-Lan Longitudinal Aging Study; ROI = region of interest; MAE = mean absolute error; VBM = voxel-based morphometry.

The overall process of the methods used is outlined in Fig. 1. This included feature set generation, brain-age prediction model construction in an independent large healthy population, and brain-age model application in a community-dwelling ILAS cohort with and without CSVD.

Feature extraction (voxel-wise and regional-wise GMV features) was conducted as follows. Before extracting individual voxel-wise GMV information via voxel-based morphometry (VBM) analytical framework, an experienced neuroradiologist (S.-C.H.) visually examined all MRI scans to exclude participants with organic brain disorders (e.g. brain tumour) or insufficient image quality scans (e.g. severe motion artefacts). Subsequently, individual voxel-wise GMV images were obtained using a standard VBM procedure with high-dimensional Diffeomorphic Anatomical Registration through Exponentiated Lie Algebra approach (DARTEL). We used the DARTEL-VBM analytical procedure in our previous studies.^4,13^ Briefly, DARTEL-VBM comprises the following (i) tissue segmentation; (ii) study-specific template generation; (iii) spatial normalization to standard Montreal Neurological Institute (MNI) space; (iv) tissue modulation for preserving the actual tissue volume after spatial warping procedure and (v) smoothed with a full width at half maximum Gaussian kernel of 6 mm. Two types of feature sets were generated from the resultant GMV images. More specifically, we applied the composite brain atlas (including 400 Schaefer’s cortical functional regions,^14^ 14 Harvard-Oxford subcortical regions^15^ and 28 spatially unbiased infratentorial template cerebellum regions)^16^ to extract the mean GMV and voxel-wise GMV of each brain region to use as the candidate feature sets. The concept of large-scale brain network is Schaefer’s cortical parcellation scheme based on. It is postulated that the human brain could be segregated into a number of large-scale functional networks comprised widely distributed regions. These interconnected brain areas under the functional network can interact to perform particular functions. Studies by functional MRI have provided evidences supporting this concept and defined seven brain networks, namely visual, somatomotor, dorsal attention, salience/ventral attention, limbic, default mode and executive control.^17^ Schaefer *et al*.^14^ then established this brain parcellation scheme according to the seven brain networks with the gradient-weighted Markov Random Field model, a hybrid model that integrates local gradient and global similarity approaches. The local gradient approach is a boundary mapping method that reflects cortical areal boundaries by detecting abrupt transitions in functional connectivity pattern. The global similarity approach uses a clustering method by assigning similar functional connectivity patterns to the same region. The resultant GMV images were eventually parcellated into 442 brain regions, which included 400 cortical regions,^14^ 14 subcortical regions^15^ and 28 cerebellum regions.^16^ We extracted the average GMV and voxel-wise GMV of each region to serve as the input features for constructing the global- and regional-level brain-age predictive models, respectively.

Brain-age prediction model construction was conducted as follows. Support vector regression (SVR) with the radial basis function kernel algorithm which was available in the Scikit-learn library was utilized to construct the global- and regional-level brain-age prediction models.^18^ For each feature set, the nested 5-fold cross-validation scheme was applied for the training data set to confirm the reliability of the constructed brain-age prediction models.^19^ Specifically, we determined the optimal C and gamma parameter of SVR algorithm from seven values (0.001, 0.01, 0.1, 1, 10, 100 and 1000) using GridSearch CV function in the inner cross-validation loop.^20^ In the outer cross-validation loop, model accuracy was evaluated by comparing brain-predicted age with chronological age via the mean absolute error (MAE) and coefficient of determination (R^2^). Subsequently, the final brain-age prediction model of the whole training data set was constructed using the optimal parameters, which were estimated using a 5-fold cross-validation scheme and then applied to the ILAS cohort to estimate the brain age of these individuals. Finally, the global-level and regional-level BAG were calculated for each participant of the ILAS cohort by subtracting the chronological age from the predicted brain age. Hence, a positive BAG indicates accelerated brain aging globally or regionally. BAG measurements were then used for further statistical analyses.

### Detection and assessment of CSVD in the ILAS cohort

Three MRI CSVD markers, CMBs, WMH and lacunes, were used to subdivide the participants of the ILAS cohort into non-CSVD and CSVD groups. First, CMBs were defined as small, rounded, or circular, well-defined, hypointense lesions within the brain parenchyma, with clear margins and ≤ 10 mm in size, on individual SWI scans.^21,22^ Microbleed mimics, such as vessels, calcification, partial volume, air-bone interfaces and haemorrhagic areas within or adjacent to an infarct were carefully excluded. Intra-rater reliability was assessed separately by evaluating CMBs in 20 randomly sampled images (K = 0.83; 95% confidence interval: 0.79–0.90). We also re-assessed CMBs in 25 randomly sampled images previously assessed by Dr. Chung and another investigator (K = 0.82; 95% confidence interval: 0.79–0.88). Second, we used quantitation method instead of visual scales to detect the WMH, which could display more sensitive and better discrimination of WMH burden.^23,24^ WMH lesion volume was estimated from T_2_-weighted FLAIR images using the previously proposed analytical pipeline, which combined with Statistical Parametric Mapping (SPM12) and the Lesion Segmentation Toolbox (version 3.0.0).^13^ In brief, the estimation pipeline first co-registered the individual T_2_-weighted FLAIR scan to the corresponding T_1_ scan and further generated WMH probability map and lesion-filling T_1_-weighted images for each participant. Using the resultant lesion-filling T_1_-weighted images with the DARTEL-VBM approach, the individual WMH probability map was spatially normalized into the standard MNI space and further modulated with the corresponding deformation field to obtain the actual WMH volume information in the MNI space. All WMH segmentations were carefully checked visually. The total lesion volume was normalized using the total intracranial volume (TIV) to calculate the WMH volume ratio for the CSVD definition. Finally, using individual T_2_-weighted FLAIR scans, lacune were manually defined as small (< 15 mm in diameter) CSF-containing cavities, located in the deep GM or white matter, with adjacent WMH.^7^

Since individual marker might also be the presentation of diseases other than CSVD, it would be a more specific strategy for CSVD definition to combine WMH with additional lesion marker, especially in the non-demented stroke-free (covert CSVD) population.^25^ In the present study, CSVD was defined as the ≥ 50th percentile of WMH volume ratio with the presence of lacunes or CMBs. The included participants from the ILAS cohort were then stratified into CSVD and non-CSVD groups.

### Measures of cognitive functions in ILAS cohort

All participants in the ILAS cohort underwent a face-to-face neuropsychological assessment administered by trained interviewers. We used the Mini-Mental State Examination (MMSE) to evaluate global cognitive performance. The performance of the four different cognitive domains was assessed using extensive neuropsychological tests. Verbal memory was determined using the delay free recall items in the Chinese Version Verbal Learning Test (CVVLT).^26^ Language function was assessed by the Boston Naming Test,^27^ visuospatial function by the copy test of the Taylor Complex Figure Test^28^ and executive function by the category (animal) verbal fluency test (VFT)^29,30^ and the Clock Drawing Test (CDT).^31,32^

### Statistical analysis

#### (1) Demographic and clinical characteristics

Statistical analyses of the demographic and clinical evaluations were conducted using IBM SPSS Statistics version 25 (IBM Corp., Armonk, NY, USA). We used the two-sample Student’s *t*-test for continuous variables and the chi-square test for categorical variables to identify statistical differences between the respective study groups. Clinical assessments of continuous variables were performed by covariance analysis (ANCOVA) after adjustment for age, sex, and years of education. Two-tailed *P*-values < 0.05, were considered statistically significant in all analyses.

#### (2) BAG comparisons between CSVD and non-CSVD groups

To identify differences in global and regional BAG between the study groups, we used ANCOVA adjusted for chronological age, the square of chronological age, sex, education years, entropy focus criterion (EFC) index, TIV and other vascular risk factors (including hypertension, diabetes, dyslipidemia and cigarette smoking status) as nuisance variables. Using individual chronological age as a nuisance variable could further adjust age bias in the brain-age predictions.^33^ The EFC index is a quantitative index for representing image blurring and ghosting induced by participants’ head movement and was calculated using the MRI Quality Control tool (https://github.com/poldracklab/mriqc).^34^ The analysis of regional BAG differences was corrected for the number of brain regions (442 brain regions, Bonferroni-corrected *P*-value < 0.05 = 0.000113, calculated by 0.05/442). No multiple comparison correction was necessary for the comparison of global BAG between the study groups, as the global BAG collapses the multivariate pattern of regional GMV into a single measure. Additionally, effect sizes were calculated using the partial eta squared (η^2^) approach.

#### (3) The mediating role of BAG in the associations between CSVD and cognitive performances

A standard mediation analysis was performed using the Mediation Toolbox developed by Tor Wager’s group (https://github.com/canlab/MediationToolbox), which has been widely used in many neuroimaging studies.^35^ We used a single-level three-variable mediation model. Mediation analysis tests whether the association between two variables can be explained by a third variable (the mediator). The hypothesis tested here was whether the global or each regional BAG (mediator, M) mediated the association between CSVD (independent variable, X) and cognitive performances (dependent variable, Y). Confounding variables as in the association analysis were regressed out in the mediation model. The significance of the mediation effect was estimated by the bias-corrected bootstrap approach (with 10 000 random samplings). More specifically, this mediation procedure could be decomposed into three regression models. The relationship between X–M and X–Y were estimated by path *a* (M = *a*X + e_m_) and path *c* (Y = *c*X + e_y_), respectively. Path *b* showed the relationship between M–Y, while accounting for the effects of X, path *c’* (Y = *b*M + *c'*X + e'_y_). The vectors e denoted residual error for regression models, respectively. Path a*b indicated the presence of a significant difference between path *c* and path *c’* (*c—c’*). All models included the confounding factors about chronological age, the square of chronological age, sex, education years, EFC index and TIV. Statistical significance was evaluated using an accelerated, bias-corrected bootstrap approach by estimating the distribution of the path coefficients by random sampling (10 000 bootstrap samples) to test each of the *a*, *b*, a*b path coefficients. We set the statistical significance threshold at *P-value* < 0.05 for all the relevant paths. The proportion of path a*b to path *c* was further calculated to evaluate the mediation effect which indicate the percentage effect of BAG can explained of CSVD to cognitive functions.

### Standard protocol approvals and patient consents

The study was approved by the Institutional Review Board of the National Yang Ming University, Taipei, Taiwan. All participants provided written informed consent.

### Data availability

The data that support the findings of this study are available from the corresponding author upon reasonable request.

## Results

### Demographics of CSVD and non-CSVD groups

Among the 760 ILAS participants with eligible brain MRI images, 9 and 17 were excluded due to incidentally detected brain tumours and head motion, respectively. A total of 734 participants were included in the analysis (Supplementary Figure 2); the histograms of their CSVD features are demonstrated in Supplementary Figure 3. The CSVD group comprised 124 participants with brain MRI showing severe WMH (≥ 50th WMH volume/TIV ratio in the total population = 0.7 × 10^−3^) in addition to the presence of any lacunes or CMBs. The non-CSVD group included 610 participants. In the CSVD group, all had severe WMH, 83 (66.9%) had lacunes, 69 (55.6%) had CMB and 28 (22.6%) had both lacunes and CMBs. The demographics of the CSVD and non-CSVD groups are presented in Table 1. Compared with the non-CSVD group, the CSVD group was significantly older, less educated and had a higher prevalence of vascular risk factors, including hypertension, diabetes mellitus and dyslipidemia. Table 1 also shows the results of the neuropsychological tests. Although all were non-demented and stroke-free (asymptomatic CSVD), participants in the CSVD group scored lower in all cognitive domains than those in the non-CSVD group, with statistical significance in the MMSE (global cognitive performance), CVVLT (verbal memory) and VFT (executive function).

**Table 1:** Comparisons of demographics and cognitive functions between CSVD and non-CSVD groups

Demographic variables	Non-CSVD	CSVD	*P*
	(*n* = 610)	(*n* = 124)	
Age (years)	61.43 ± 7.82	69.07 ± 9.07	<0.001^a^
Sex (male/female)	264/346	61/63	0.227^b^
Education years	7.53 ± 5.09	4.88 ± 4.99	<0.00^a^
EFC index	0.544 ± 0.023	0.546 ± 0.022	0.610^a^
TIV (litre)	1.310 ± 0.119	1.324 ± 0.123	0.222^a^
**Vascular risk factors**			
Hypertension	186 (30.5%)	58 (46.8%)	<0.00^b^
Diabetes mellitus	70 (11.5%)	31 (25.0%)	<0.00^b^
Dyslipidemia	29 (4.8%)	12 (9.7%)	0.030^b^
Smoking	86 (14.1%)	21 (16.9%)	0.058^b^
**Cognitive performance**			
Mini-Mental State Examination	26.7 ± 3.1	24.2 ± 4.9	0.002^c^
10 min CVVLT	6.8 ± 1.9	5.5 ± 2.3	0.006^c^
Clock drawing test	8.1 ± 2.2	6.8 ± 2.9	0.147^c^
Taylor complex figure test	31.5 ± 5.7	27.9 ± 8.9	0.088^c^
Boston naming test	12.5 ± 2.4	11.3 ± 2.5	0.851^c^
Verbal fluency test	15.3 ± 4.9	13.2 ± 4.4	0.018^c^
Backward digit test	3.8 ± 2.0	2.8 ± 2.1	0.423^c^

CSVD = cerebral small vessel disease; CVVLT = Chinese version Verbal Learning Test; EFC, entropy focus criterion; TIV, total intracranial volume

^a^ Two-sample *t*-test analysis.

^b^ Two-group χ^2^ test.

^c^ Two-group analysis of covariance adjusted for age, sex and education years.

### Performance of brain-age prediction model in the training data set

**Figure 2:**
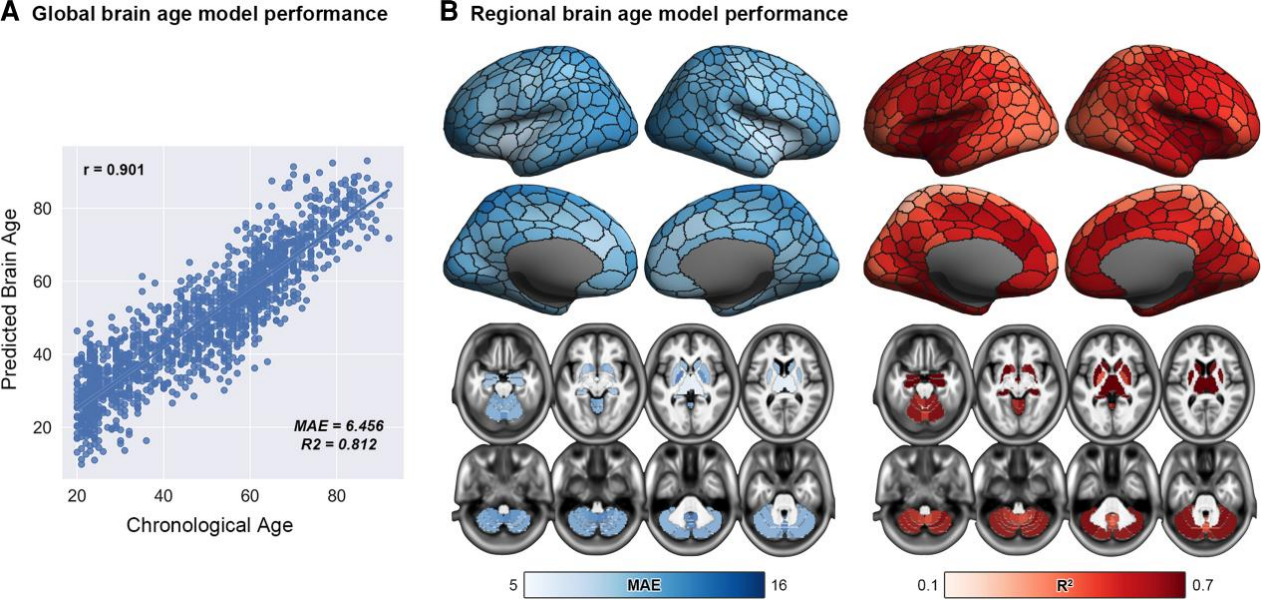
**Performances of global and regional brain-age prediction models in the training data set.** (**A**) Global brain-predicted age was highly associated with chronological age (*r* = 0.901, *P* < 0.001; MAE = 6.456 years and R^2^ = 0.812). (**B**) Regional brain-age prediction models also demonstrated moderate to good prediction (MAE range from 5.772 to 13.598 years and R^2^ range from 0.234 to 0.847). MAE = mean absolute error; *r* = correlation coefficient; R^2^ = coefficient of determination.

We demonstrated the association between chronological age and global brain-predicted age within the independent training data set (Fig. 2A). As expected, global brain-predicted age was highly associated with chronological age (r = 0.901, *P* < 0.001; R^2^ = 0.812, and MAE = 6.456 years). Furthermore, the regional brain-age prediction model also demonstrated moderate to good prediction, as illustrated in Fig. 2B (R^2^ ranges from 0.234 to 0.847, and MAE ranges from 5.772 to 13.598 years).

### Global and regional brain ages in the CSVD and non-CSVD groups

**Figure 3:**
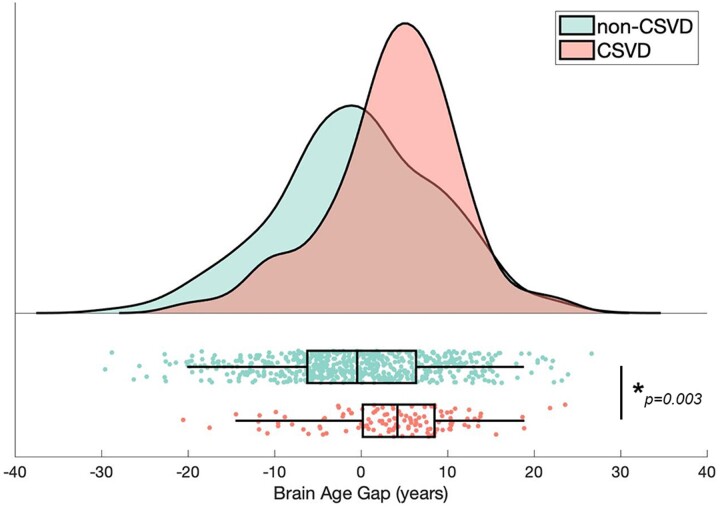
**Comparison of global BAG between the CSVD and non-CSVD groups.** The plot shows distribution (probability density plot), summary data (box plot) and raw observations of the global BAG for the CSVD and non-CSVD groups. ANCOVA test adjusted for chronological age, the square of chronological age, sex, education years, EFC index, TIV and vascular risk factors (including hypertension, diabetes, dyslipidemia, and cigarette smoking status) was used. The estimated global BAG was significantly greater in the CSVD group (BAG = 3.71 ± 7.60) than the non-CSVD group (BAG=−0.43 ± 9.47; *P* = 0.003, η^2^ = 0.012). *P < 0.05. BAG = brain-age gap; CSVD = cerebral small vessel disease.

The results showed that the estimated global BAG was significantly increased in the CSVD group (BAG = 3.71 ± 7.60) than the non-CSVD group (BAG=−0.43 ± 9.47) after adjusting for confounders including chronological age, sex, education years, TIV, EFC and the presence of vascular risk factors including hypertension, diabetes mellitus, dyslipidemia and cigarette smoking (*P* = 0.003, η^2^ = 0.012, Fig. 3).

**Figure 4:**
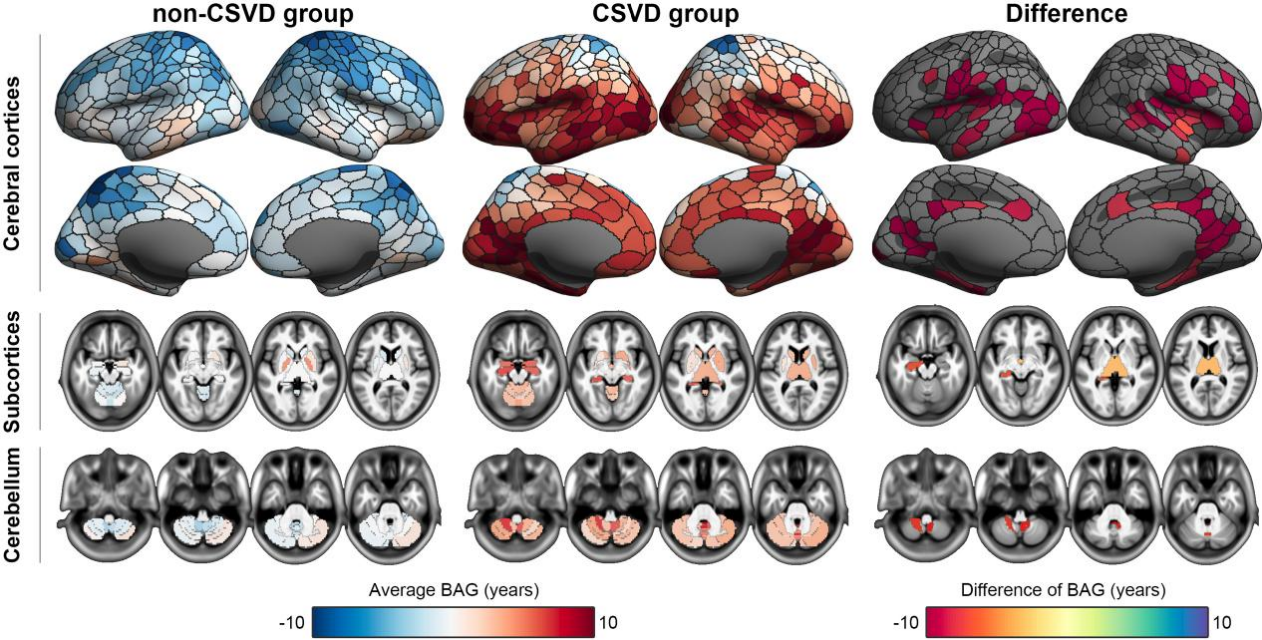
**Comparison of regional BAGs between CSVD and non-CSVD groups.** The left and middle columns show the averaged BAGs of 442 brain regions in non-CSVD and CSVD groups. The right column shows the regions with significant BAG differences between CSVD and non-CSVD groups. Statistical significance was set as *P* < 0.000113 (Bonferroni correction). BAG = brain-age gap; CSVD = cerebral small vessel disease.

**Figure 5:**
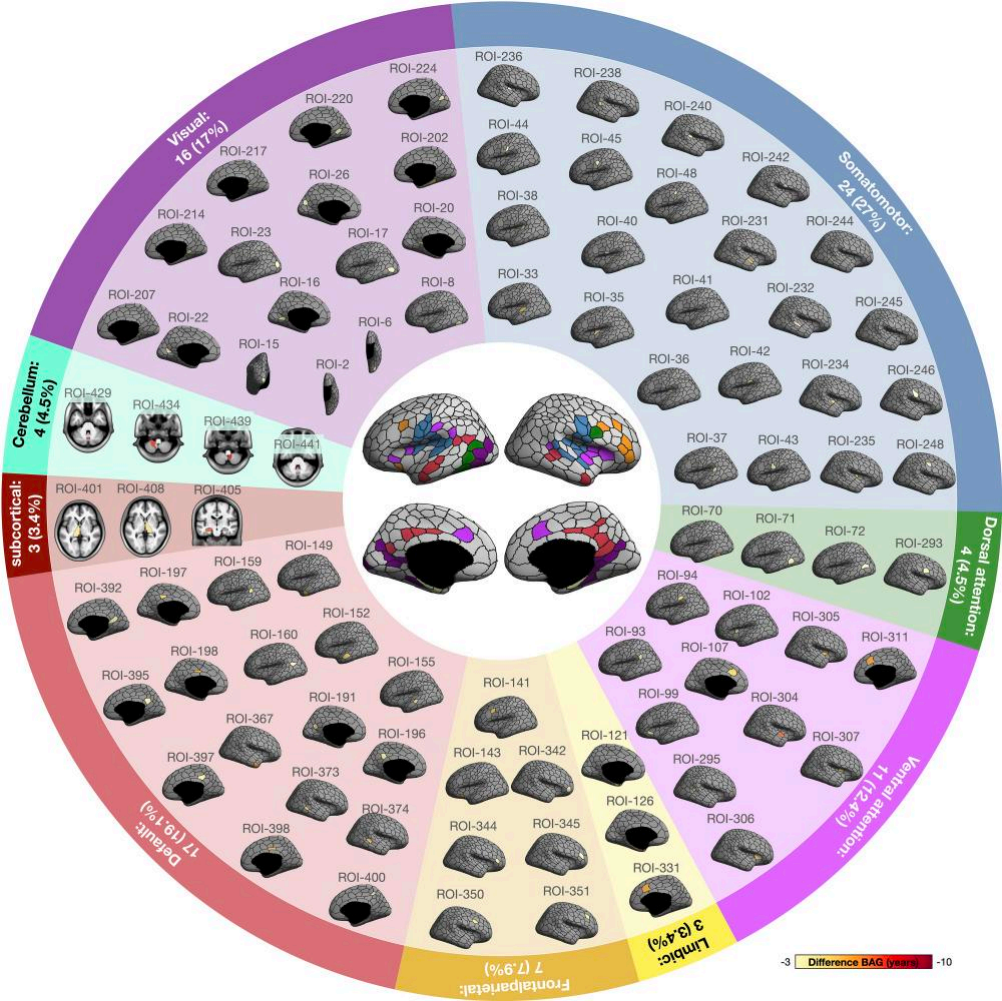
**Demonstration of 89 brain regions with advanced regional brain aging in the CSVD group and the networks the 89 regions belonged to.** Numbers (%) at the outermost circle represent the distribution of networks among the 89 brain regions. BAG = brain-age gap; CSVD = cerebral small vessel disease.

Furthermore, using the regional-level brain-predicted age models, we also demonstrated the regional-specific advanced aging profile in the CSVD group. After adjusting for confounders and the Bonferroni correction, 89 brain regions showed significantly different BAG between the CSVD and non-CSVD groups (Fig. 4). These regions included 82 cerebral cortical regions (mainly fronto-temporal cortices alongside the Sylvian fissure and the posterior cingulate cortex), three subcortical regions (left and right thalamus in addition to the left hippocampus) and four regions in the cerebellum (Fig. 5). Mapping these 89 regions with the relevant brain networks showed that all seven brain networks were involved (Fig. 5). Of these, cortical regions with significantly increased BAG in CSVD mostly belonged to the somatomotor and default mode networks (Fig. 5). The mean differences between the groups in these 89 regions are shown in Fig. 5 and Supplementary Table 2.

### Mediating role of BAG in the associations between CSVD and cognitive functions

**Figure 6:**
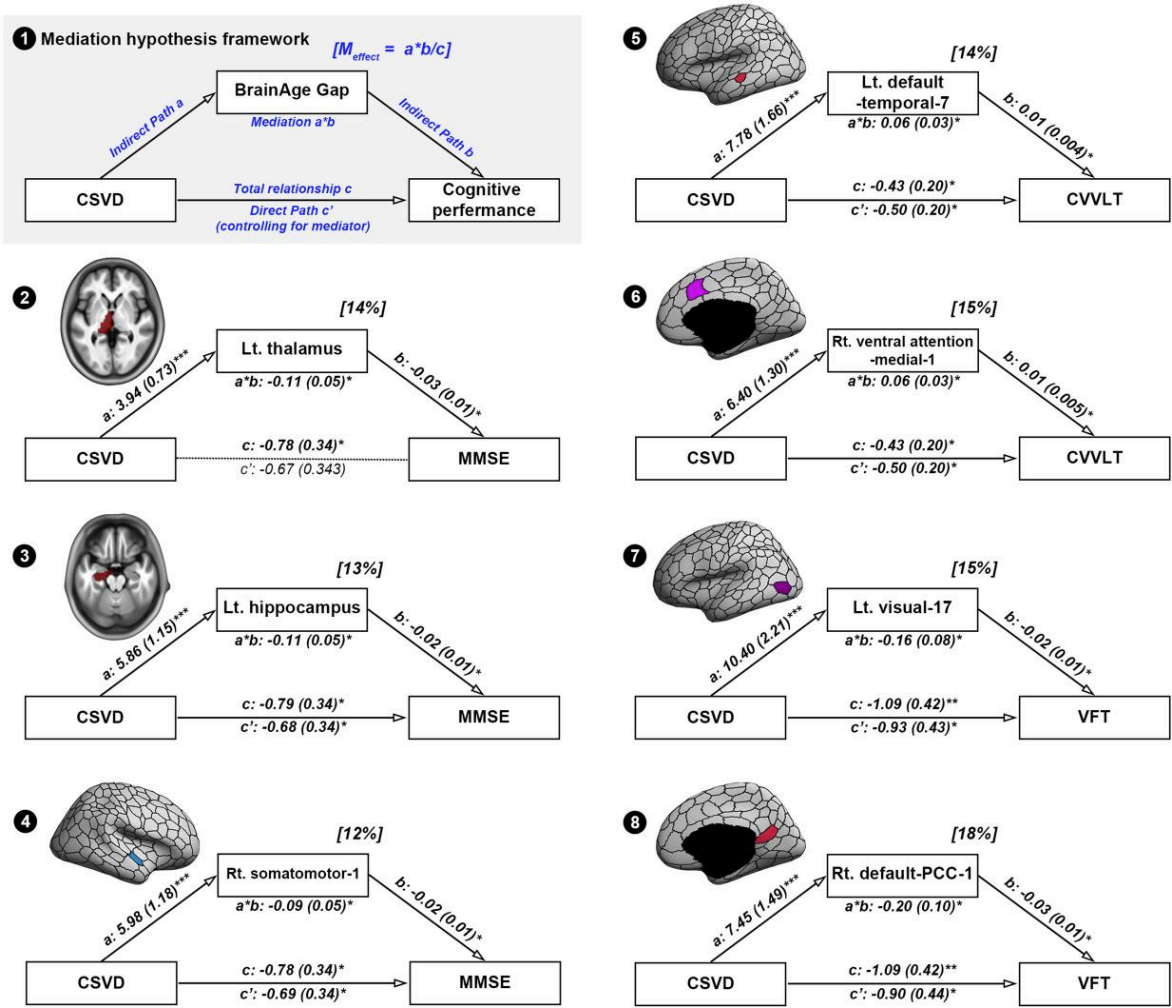
**The results of mediation analyses.** (1) The diagram of mediation hypothesis framework. (2–8) Seven regional BAGs as potential mediators between CSVD and related cognitive impairments. The path coefficients and mediating magnitude (effect) are provided in each model. Path a: the effect of the CSVD on the mediator (regional BAG); Path b: the effect of the mediator (regional BAG) on the cognitive test’s score; Path c: the effect of CSVD on the cognitive test’s score; Path c’: the direct effect of CSVD on the cognitive score controlling for the mediator (regional BAG); Path a*b: the difference and its significance between path c and c’. Statistical significance of the mediating effect was evaluated with a bootstrap test. The dark solid and light dashed lines indicate a significant and non-significant relationship between each variable, respectively. Numbers are the corresponding mean path coefficients with standard error in brackets. Percent values above mediator indicate the effect size calculated from the proportion between path a*b and path c. *P < 0.05; **P < 0.01; ***P < 0.001. BAG = brain-age gap; CSVD = cerebral small vessel disease; CVVLT = Chinese Version Verbal Learning Test; MMSE = Mini-Mental State Examination; VFT = verbal fluency test.

We next investigated whether advanced brain aging, globally and regionally, in the CSVD group, is related to cognitive impairment, namely, global cognitive performance (MMSE), verbal memory (CVVLT) and executive function. Figure 6 displays path diagrams for the interactions of these variables in a mediation framework. The results showed that global BAG failed to mediate the relationship between CSVD and any related cognitive impairments. In contrast, of the 89 regions with significantly increased BAG in the CSVD group, seven regional BAGs showed significant mediation effects in CSVD-related cognitive impairment (Fig. 6). Regional BAG in one region of the somatomotor network (mediation magnitude: 12% in MMSE), two regions of the default mode network (mediation magnitude: 14% in CVVLT and 18% in VFT), one region of the visual network (mediation magnitude: 15% in VFT), and one region of the ventral attention network (mediation magnitude: 15% in CVVLT) significantly explained the cognitive function of specific domains in CSVD (Fig. 6). There were also two regional BAGs in the subcortical areas with significant mediation effects between CSVD and global cognitive performance (MMSE); regional BAG in the left hippocampus (mediation magnitude: 13%) and left thalamus (mediation magnitude: 14%) significantly explained the poorer global cognitive performance in CSVD (Fig. 6). The detailed data of the path coefficients, significance and magnitude (amount of changes) of mediation effects for MMSE, CVVLT and VFT in 89 regional BAG is provided in Supplementary Table 3–5. We set the statistical significance *P* < 0.05 uncorrected in 89 mediation models. These seven results would not survive if family-wise error rate (FWER) corrected.

## Discussion

The main findings of this study were as follows. First, adults aged ≥ 50 years with asymptomatic CSVD showed advanced global and regional brain ages, estimated with GM features, compared with participants without CSVD. The average BAG (difference between predicted brain age and chronological age) was more than 3 years globally and 4–13 years within certain brain regions. Second, the regional rather than global BAG mediated the associations between CSVD and related cognitive impairments. Notably, the significance of mediation effects did not survive if FWER corrected (*P* < 0.05/89).

The human brain continuously changes throughout the lifespan. Brain volume begins to shrink from the fourth decade of life.^36,37^ To determine whether an individual’s brain structural changes deviate from the normal aging process and thus how certain diseases exert their effects on the aging brain, neuroimaging-based brain-age prediction is an emerging and promising approach in neuroscience research.^3,4,33^ These studies have shown advanced brain age in several neurological or systemic diseases and that the BAG can predict the risk of neurodegenerative diseases and mortality in the elderly.^2,3,38-42^ There are other large cohort studies revealing a relationship between the predicted brain age and WMH volume.^38,43^ They also found a positive association between predicted brain age or BAG and WMH volume. However, in their approaches, confounders such as age or vascular risk factors were not corrected and other CSVD markers were not considered. We are the first to validate the associations between CSVD and predicted brain age. Our results suggest that CSVD, even when asymptomatic, can influence the neurobiology of global brain aging, independent of chronological age and vascular risk factors. The present study may contribute insights to the underlying mechanisms, not only of the CSVD-related clinical consequences, but also of CSVD-associated neurodegenerative diseases.^44,45^ Our results also suggest that CSVD should be considered in future studies that aim to evaluate abnormal brain aging in other diseases.

CSVD is manifested by focal brain lesions but usually affects remote and widespread brain functions and structures beyond the local lesions.^13,46,47^ In addition to detected structural or functional abnormalities in normal-appearance white matter surrounding the CSVD lesions,^48,49^ increasing evidence suggests that CSVD, though presented with local lesions mostly located in white matter, is a global disease that disrupts the brain functional networks.^46,50^ The functional networks refer to different brain regions that are spatially remote but functionally linked to maintain brain functions.^17^ Abnormal networks that have been reported in CSVD include the default mode network, the dorsal attention network and the frontoparietal control network.^46,50^ It has been proposed that CSVD lesions located in white matter or rich-club nodes would disconnect the nerve tract of networks and result in structural changes, commonly volume reduction, in remote cortical regions within the networks.^46^ CSVD may also cause advanced brain aging via this postulated pathway. In the present study, we used network-based brain parcellation to measure regional brain age to understand the spatial effects of CSVD on brain age.^14^ We investigated whether CSVD at the early, asymptomatic stage had accelerated brain age in regions restricted to certain networks and attempted to determine the early network involved in the pathophysiology of CSVD. Nevertheless, the results showed that, in asymptomatic CSVD, all networks were involved in the accelerating brain age process, with the two most involved regions being the somatomotor network and the default mode network.

Although advanced brain age in CSVD was noted globally and in 89 brain regions, only seven cortical and subcortical regions were associated with CSVD-related cognitive functions. Our results suggest that region-specific brain age estimations might provide more information than the global brain age as a measure of brain integrity. These regions might be strategic advanced brain age in cognitive impairment, e.g. accelerating aging involving specific sites that are critical for cognitive functions. Among these strategic regions, the left thalamus significantly mediated the effect of CSVD according to global cognitive performance (MMSE). Thalamus is an important node in several networks that connect with many remote cortical, subcortical and cerebellar regions.^51-53^ It integrates neural networks involving cognitive functions including processes of attention, speed of information processing and working memory.^53^ Strategically involved thalamus has also been noted in age-related cognitive impairment and post-stroke dementia.^53,54^ Functional mapping studies have shown functional involvement of the thalamus with several networks including somatomotor, default mode, dorsal attention, and visual and frontoparietal networks.^51-53^ Whether and how accelerating brain age in the thalamus mediates CSVD-related cognitive impairment through these brain networks requires further investigation.

Another brain region showing advanced aging and association with global cognitive function in CSVD is the left hippocampus. Hippocampus is in responsible for learning and memory; and its structural or functional impairments are involved early in several neurodegenerative diseases.^55^ Recent literatures have revealed that blood–brain barrier (BBB) breakdown might be the earliest neurovascular dysfunction involved in the aging and neurodegenerative brains.^56-58^ These studies consistently indicate BBB dysfunction in the hippocampus an early event and contributing to cognitive impairment in the aging and neurodegenerative processes. We have found advanced aging in the left hippocampus in early stage of CSVD. Furthermore, left hippocampus regional BAG showed significant mediation effect between CSVD and global cognitive function. The present findings have provided additional evidences supporting hippocampus a critical region where neurovascular dysfunction (CSVD) and neurodegeneration intertwine and lead to cognitive impairments in the elderly.

Supporting the global involvement of CSVD revealed by brain structural and network studies, a recent systemic review also demonstrated that CSVD may affect all kinds of cognitive domains in both asymptomatic and stroke or demented populations.^59^ Several studies have also revealed heterogeneous cognitive profiles among individuals with similar degrees of CSVD on MRI.^5,59^ The present study showed that increased BAG in seven regions was significantly mediating cognitive impairment of respective domains in CSVD. These results suggest that advanced brain age in these specific regions may have distinct cognitive effects in CSVD. We postulated that the different involvement or extent of accelerating brain age in these strategic regions may explain, at least in part, the variability in cognitive symptoms among individuals with CSVD. BAG in these regions may be a suitable clinical biomarker for predicting cognitive outcomes in patients with CSVD. Further validation in other cohorts with longitudinal follow-ups is warranted.

The following methodological considerations should be considered when interpreting the current results. Using a relatively large sample size to achieve a higher prediction performance of the brain-age prediction model, one potential limitation is the source variability of the training data sets. As the inclusion criteria were different for each data set, the constructed prediction model may suffer from some potential bias. However, we not only visually examined all MRI scans but also screened these individuals based on various criteria to ensure that they were free of neuropsychiatric diseases, history of head trauma or other major medical issues. Furthermore, the present model of brain-age prediction only used GM features (especially GMV) from T_1_w MRI. Future research with other brain features from modalities such as white matter microstructural integrity from diffusion tensor imaging and network or/and hemodynamic profiles from resting-state functional MRI (rs-fMRI) would improve our understanding of the effects of CSVD on brain aging. Regarding the study population, our CSVD participants were community-based non-stroke and non-demented middle-to-old adults with a relatively milder degree of CSVD lesions. This setting aimed to determine the early pathophysiology of CSVD. However, it may also limit the generalizability of our findings to more severe CSVD. In the cognitive assessments, we included several neuropsychological tests since CSVD has been shown affecting all major cognitive domains.^59^ However, the number of tests used to indicate one cognitive domain might not be optimal. Additionally, the present study did not consider the hereditary CSVD, such as cerebral autosomal dominant arteriopathy with subcortical infarcts and leukoencephalopathy and other rare aetiologies of CSVD in our population. In the exploratory analyses about the mediating effects of regional BAGs in CSVD-associated cognitive domains, we set the statistical significance *P* < 0.05 uncorrected in 89 mediation models. More analyses would be needed to validate these results due to the potential type I error. The present study defined CSVD as as the ≥ 50th percentile of WMH volume ratio with the presence of lacunes or CMBs and analyzed using a dichotomous variable, e.g. CSVD and non-CSVD groups. This method setting could provide clearer clinical implications. However, other methods which use continuous neuroimaging variables to represent CSVD^60^ might more accurately capture degrees of CSVD burden and offer greater statistical power. It would be a research priority to investigate the most optimal way to define and analyze CSVD in future. Finally, the present study analyzed the spatial characteristics but not the temporal relationship of brain age in CSVD and its related cognitive impairments. Longitudinal studies or other specific computational analyses are needed to elucidate the regional order of abnormal brain aging and their causal relationships in CSVD.

In conclusion, our study revealed evidence of accelerated brain aging in asymptomatic CSVD. The spatial distributions of advanced brain age in CSVD were mainly in the fronto-temporal cortices alongside the Sylvian fissure, the posterior cingulate cortex, bilateral thalamus, and left hippocampus, in which all networks were involved. We also showed the interactive effects between CSVD and accelerated brain age and found region-specific mediation effects of BAG in the relationship between CSVD and related cognitive impairments. Our results may contribute insights into the mechanisms underlying the effects of CSVD on brain aging and the associated cognitive impairment.

## Supplementary Material

fcac233_Supplementary_Data
