# Supplementary material for: Regional rather than global brain age mediates cognitive function in cerebral small vessel disease
Source: Brain Commun. 2022 Sep 14;4(5):fcac233. doi: 10.1093/braincomms/fcac233 (PMC9525017; doi:10.1093/braincomms/fcac233)

Supplementary Table 1 Profiles of dataset for brain-age prediction model construction

Cohort	N	Age<br>Years, mean±SD [range]	Sex<br>men/women	Scanner	Coil	Sequence	Protocol parameters
National Yang Ming Chiao Tung University	808	46.2±20.4 [18-92]	407/401	Siemens Tim Trio (3T)	12-channel / 32-channel phased-array head coil	MPRAGE	TR/ TE/ TI = 3500/3.5/ 1100 msec; flip angle = 7°; NEX = 1; FOV = 256 x 256 mm^2; matrix size = 256 x 256; 192 sagittal slices; and voxel size = 1.0 x 1.0 x 1.0 mm^3
Taipei Veterans General Hospital	293	54.4±17.8 [22-92]	94/199	GE Discovery MR750 (3T)	8-channel phased-array head coil	IR-FSPGR	TR/TE/TI = 9.4/4.0/450 msec; flip angle = 12°; NEX = 1; FOV = 256 x 256 mm^2; matrix size = 256 x 256; 172 axial slices; and voxel size = 1.0 x 1.0 x 1.0 mm^3
						BRAVO	TR/TE/TI = 9.2/3.7/450 msec; flip angle = 12°; NEX = 1; FOV = 256 x 256 mm^2; matrix size = 256 x 256; 168 axial slices; and voxel size = 1.0 x 1.0 x 1.0 mm^3
Tri-Service General Hospital	68	42.0±11.1 [21-62]	21/47	GE Discovery MR750 (3T)	8-channel phased-array head coil	BRAVO	TR/TE/TI = 10.17/4.16/450 msec; flip angle = 12°; NEX = 1; FOV = 256 × 256 mm^2; matrix size = 256 × 256; 172 axial slices; and voxel size = 1.0 x 1.0 x 1.0 mm^3
Kaohsiung Chang Gung Memorial Hospital	244	48.5±14.3 [20-75]	127/117	Siemens Skyra (3T)	20-channel phased-array head/neck coil	MPRAGE	TR/ TE/TI = 1800/2.7/900 msec; flip angle = 9°; NEX = 1; FOV = 230 x 230 mm^2; matrix size = 192 x 192; 144 sagittal slices; and voxel size = 1.2 x 1.2 x 1.2 mm^3
				GE Signa Excite (3T)	8-channel phased-array head coil	IR-FSPGR	TR/ TE/ TI = 9.5/3.9/450 msec; flip angle = 15°; NEX = 1; FOV = 240 × 240 mm^2; matrix size = 512 × 512; 110 axial slices; and voxel size = 0.47 × 0.47 × 1.3 mm^3
Keelung Chang Gung Memorial Hospital	69	65.0±5.1 [52-77]	32/37	Siemens Skyra (3T)	20-channel phased-array head/neck coil	MPRAGE	TR/TE/TI = 2,200/2.45/900 msec; flip angle = 8°, NEX=1; FOV = 256 x 256 mm^2; matrix size = 256 x 256; 176 sagittal slices; and voxel size = 1.0 x 1.0 x 1.0 mm^3

TR = Repetition time; TE = echo time; TI = inversion time; NEX = number of excitations; FOV = field of view.

Supplementary Table 2. ROIs with BAG change in CSVD patients compared with healthy controls

ROI Index	Region	Network	Difference of BAG	F-value	p-value	Effect size (η_p^2)
306	RH_SalVentAttn_FrOper_5	Ventral Attention	9.24	33.07	0.00000001	0.044
220	RH_Vis_20	Visual	11.58	30.73	0.00000004	0.041
8	LH_Vis_8	Visual	11.75	30.26	0.00000005	0.040
22	LH_Vis_22	Visual	11.28	29.56	0.00000007	0.039
236	RH_SomMot_6	Somatomotor	11.59	27.76	0.00000018	0.037
44	LH_SomMot_13	Somatomotor	10.75	26.05	0.00000043	0.035
71	LH_DorsAttn_Post_3	Dorsal Attention	12.28	25.95	0.00000045	0.035
72	LH_DorsAttn_Post_4	Dorsal Attention	11.31	25.42	0.00000058	0.034
245	RH_SomMot_15	Somatomotor	12.82	24.74	0.00000082	0.033
70	LH_DorsAttn_Post_2	Dorsal Attention	8.57	24.70	0.00000084	0.033
397	RH_Default_PCC_6	Default	9.56	24.09	0.00000114	0.032
367	RH_Default_Temp_1	Default	7.42	23.64	0.00000143	0.032
374	RH_Default_Temp_8	Default	8.73	23.44	0.00000158	0.031
6	LH_Vis_6	Visual	11.06	23.21	0.00000177	0.031
160	LH_Default_Temp_12	Default	11.36	23.04	0.00000193	0.031
392	RH_Default_PCC_1	Default	10.18	23.01	0.00000195	0.031
240	RH_SomMot_10	Somatomotor	11.21	22.69	0.00000230	0.031
214	RH_Vis_14	Visual	9.67	22.67	0.00000233	0.030
224	RH_Vis_24	Visual	11.71	22.18	0.00000298	0.030
232	RH_SomMot_2	Somatomotor	9.98	22.13	0.00000306	0.030
16	LH_Vis_16	Visual	9.64	21.43	0.00000435	0.029
351	RH_Cont_PFCl_11	Frontalparietal	9.18	21.11	0.00000512	0.028
41	LH_SomMot_10	Somatomotor	11.22	20.88	0.00000575	0.028
246	RH_SomMot_16	Somatomotor	10.81	20.69	0.00000635	0.028
293	RH_DorsAttn_PrCv_1	Dorsal Attention	9.67	20.52	0.00000690	0.028
152	LH_Default_Temp_4	Default	8.88	20.39	0.00000737	0.028
401	Left_Thalamus	Subcortical	4.08	20.39	0.00000737	0.028
405	Left_Hippocampus	Subcortical	8.07	20.29	0.00000776	0.027
36	LH_SomMot_5	Somatomotor	9.87	20.27	0.00000785	0.027
38	LH_SomMot_7	Somatomotor	11.79	19.90	0.00000947	0.027
305	RH_SalVentAttn_FrOper_4	Ventral Attention	7.97	19.78	0.00001008	0.027
234	RH_SomMot_4	Somatomotor	8.93	19.72	0.00001036	0.027
439	Right_Cerebellum_IX	Cerebellum	9.68	19.64	0.00001079	0.027
235	RH_SomMot_5	Somatomotor	8.70	19.50	0.00001162	0.026
102	LH_SalVentAttn_FrOper_6	Ventral Attention	9.34	19.44	0.00001197	0.026
345	RH_Cont_PFCl_5	Frontalparietal	10.10	19.18	0.00001367	0.026
238	RH_SomMot_8	Somatomotor	9.90	19.15	0.00001385	0.026
196	LH_Default_PCC_7	Default	9.70	19.14	0.00001391	0.026
434	Left_Cerebellum_VIIIb	Cerebellum	8.87	19.02	0.00001479	0.026
373	RH_Default_Temp_7	Default	8.50	18.89	0.00001587	0.026
20	LH_Vis_20	Visual	9.95	18.86	0.00001610	0.025
155	LH_Default_Temp_7	Default	9.05	18.84	0.00001626	0.025
242	RH_SomMot_12	Somatomotor	10.88	18.82	0.00001639	0.025
107	LH_SalVentAttn_Med_1	Ventral Attention	8.63	18.76	0.00001690	0.025
344	RH_Cont_PFCl_4	Frontalparietal	9.48	18.58	0.00001854	0.025
149	LH_Default_Temp_1	Default	8.10	18.40	0.00002037	0.025

331	RH_Limbic_TempPole_7	Limbic	7.63	18.38	0.00002054	0.025
350	RH_Cont_PFCl_10	Frontalparietal	8.78	18.25	0.00002196	0.025
408	Right_Thalamus	Subcortical	4.18	18.05	0.00002437	0.024
17	LH_Vis_17	Visual	12.62	17.85	0.00002691	0.024
40	LH_SomMot_9	Somatomotor	10.21	17.83	0.00002728	0.024
311	RH_SalVentAttn_Med_1	Ventral Attention	7.80	17.78	0.00002796	0.024
43	LH_SomMot_12	Somatomotor	10.01	17.71	0.00002893	0.024
207	RH_Vis_7	Visual	7.94	17.71	0.00002897	0.024
441	Cerebellum_Vermis_X	Cerebellum	13.86	17.71	0.00002897	0.024
400	RH_Default_PCC_9	Default	10.58	17.64	0.00003011	0.024
307	RH_SalVentAttn_FrOper_6	Ventral Attention	8.65	17.56	0.00003125	0.024
342	RH_Cont_PFCl_2	Frontalparietal	10.31	17.55	0.00003151	0.024
35	LH_SomMot_4	Somatomotor	9.08	17.41	0.00003384	0.024
42	LH_SomMot_11	Somatomotor	10.46	17.18	0.00003805	0.023
126	LH_Limbic_TempPole_8	Limbic	8.25	17.01	0.00004157	0.023
197	LH_Default_PCC_8	Default	8.73	16.99	0.00004201	0.023
202	RH_Vis_2	Visual	8.29	16.98	0.00004209	0.023
141	LH_Cont_PFCl_8	Frontalparietal	8.44	16.91	0.00004376	0.023
143	LH_Cont_PFCv_1	Frontalparietal	7.35	16.80	0.00004637	0.023
99	LH_SalVentAttn_FrOper_3	Ventral Attention	9.98	16.75	0.00004744	0.023
121	LH_Limbic_TempPole_3	Limbic	9.58	16.72	0.00004814	0.023
26	LH_Vis_26	Visual	10.87	16.68	0.00004910	0.023
248	RH_SomMot_18	Somatomotor	9.49	16.39	0.00005708	0.022
2	LH_Vis_2	Visual	8.56	16.23	0.00006205	0.022
48	LH_SomMot_17	Somatomotor	9.93	16.17	0.00006415	0.022
244	RH_SomMot_14	Somatomotor	10.18	16.15	0.00006470	0.022
429	Cerebellum_Vermis_VIIb	Cerebellum	9.10	16.05	0.00006825	0.022
217	RH_Vis_17	Visual	8.69	15.98	0.00007044	0.022
15	LH_Vis_15	Visual	11.15	15.96	0.00007139	0.022
231	RH_SomMot_1	Somatomotor	7.92	15.94	0.00007198	0.022
33	LH_SomMot_2	Somatomotor	8.46	15.86	0.00007503	0.022
191	LH_Default_PCC_2	Default	9.61	15.65	0.00008359	0.021
295	RH_SalVentAttn_TempOccPar_2	Ventral Attention	8.76	15.64	0.00008399	0.021
45	LH_SomMot_14	Somatomotor	10.23	15.60	0.00008595	0.021
304	RH_SalVentAttn_FrOper_3	Ventral Attention	6.63	15.60	0.00008603	0.021
398	RH_Default_PCC_7	Default	7.74	15.59	0.00008624	0.021
198	LH_Default_PCC_9	Default	7.82	15.46	0.00009239	0.021
93	LH_SalVentAttn_ParOper_2	Ventral Attention	9.74	15.46	0.00009259	0.021
94	LH_SalVentAttn_ParOper_3	Ventral Attention	8.83	15.35	0.00009797	0.021
37	LH_SomMot_6	Somatomotor	10.76	15.23	0.00010387	0.021
23	LH_Vis_23	Visual	10.75	15.15	0.00010838	0.021
159	LH_Default_Temp_11	Default	9.42	15.09	0.00011172	0.021
395	RH_Default_PCC_4	Default	10.07	15.09	0.00011173	0.021
275	RH_DorsAttn_Post_5	Dorsal Attention	9.52	15.00	0.00011720	0.020
206	RH_Vis_6	Visual	6.49	14.99	0.00011769	0.020
167	LH_Default_PFC_2	Default	10.82	14.84	0.00012738	0.020
134	LH_Cont_PFCl_1	Frontalparietal	10.33	14.82	0.00012863	0.020
137	LH_Cont_PFCl_4	Frontalparietal	10.34	14.81	0.00012974	0.020
252	RH_SomMot_22	Somatomotor	9.58	14.74	0.00013459	0.020
201	RH_Vis_1	Visual	6.40	14.72	0.00013583	0.020

340	RH_Cont_PFCv_1	Frontalparietal	7.51	14.72	0.00013594	0.020
92	LH_SalVentAttn_ParOper_1	Ventral Attention	10.15	14.65	0.00014083	0.020
313	RH_SalVentAttn_Med_3	Ventral Attention	8.11	14.62	0.00014255	0.020
18	LH_Vis_18	Visual	14.78	14.61	0.00014379	0.020
378	RH_Default_PFCv_4	Default	6.97	14.60	0.00014432	0.020
123	LH_Limbic_TempPole_5	Limbic	8.30	14.54	0.00014921	0.020
170	LH_Default_PFC_5	Default	8.94	14.53	0.00014966	0.020
219	RH_Vis_19	Visual	10.94	14.50	0.00015195	0.020
100	LH_SalVentAttn_FrOper_4	Ventral Attention	6.90	14.35	0.00016460	0.020
349	RH_Cont_PFCl_9	Frontalparietal	8.57	14.33	0.00016658	0.019
346	RH_Cont_PFCl_6	Frontalparietal	9.55	14.31	0.00016767	0.019
163	LH_Default_Temp_15	Default	8.35	14.16	0.00018137	0.019
343	RH_Cont_PFCl_3	Frontalparietal	10.06	13.93	0.00020428	0.019
358	RH_Cont_Cing_1	Frontalparietal	8.46	13.89	0.00020872	0.019
360	RH_Cont_PFCmp_1	Frontalparietal	7.90	13.87	0.00021167	0.019
3	LH_Vis_3	Visual	6.16	13.74	0.00022601	0.019
237	RH_SomMot_7	Somatomotor	9.08	13.71	0.00022997	0.019
120	LH_Limbic_TempPole_2	Limbic	9.05	13.40	0.00027034	0.018
13	LH_Vis_13	Visual	6.44	13.35	0.00027719	0.018
423	Cerebellum_Vermis_CrusI	Cerebellum	9.00	13.27	0.00028886	0.018
101	LH_SalVentAttn_FrOper_5	Ventral Attention	8.04	13.21	0.00029832	0.018
309	RH_SalVentAttn_FrOper_8	Ventral Attention	8.27	13.08	0.00031873	0.018
368	RH_Default_Temp_2	Default	5.66	13.06	0.00032242	0.018
46	LH_SomMot_15	Somatomotor	7.74	13.03	0.00032833	0.018
166	LH_Default_PFC_1	Default	7.50	13.03	0.00032840	0.018
50	LH_SomMot_19	Somatomotor	9.54	12.90	0.00035065	0.018
301	RH_SalVentAttn_PrC_1	Ventral Attention	6.94	12.88	0.00035525	0.018
190	LH_Default_PCC_1	Default	9.50	12.82	0.00036504	0.017
308	RH_SalVentAttn_FrOper_7	Ventral Attention	7.92	12.81	0.00036762	0.017
39	LH_SomMot_8	Somatomotor	8.75	12.77	0.00037507	0.017
4	LH_Vis_4	Visual	6.90	12.77	0.00037541	0.017
294	RH_SalVentAttn_TempOccPar_1	Ventral Attention	6.82	12.77	0.00037580	0.017
399	RH_Default_PCC_8	Default	9.97	12.76	0.00037703	0.017
203	RH_Vis_3	Visual	6.00	12.70	0.00039006	0.017
171	LH_Default_PFC_6	Default	12.42	12.68	0.00039431	0.017
111	LH_SalVentAttn_Med_5	Ventral Attention	9.05	12.67	0.00039511	0.017
412	Right_Hippocampus	Subcortical	6.90	12.42	0.00045138	0.017
319	RH_Limbic_OFC_1	Limbic	7.05	12.38	0.00046090	0.017
200	LH_Default_PCC_11	Default	7.67	12.15	0.00052079	0.017
34	LH_SomMot_3	Somatomotor	8.70	12.14	0.00052423	0.017
205	RH_Vis_5	Visual	6.07	12.09	0.00053654	0.016
440	Left_Cerebellum_X	Cerebellum	6.48	12.02	0.00055716	0.016
432	Cerebellum_Vermis_VIIIa	Cerebellum	8.25	12.00	0.00056234	0.016
138	LH_Cont_PFCl_5	Frontalparietal	10.58	11.95	0.00057798	0.016
363	RH_Default_Par_2	Default	8.75	11.95	0.00057867	0.016
250	RH_SomMot_20	Somatomotor	7.31	11.95	0.00057934	0.016
174	LH_Default_PFC_9	Default	9.00	11.84	0.00061439	0.016
233	RH_SomMot_3	Somatomotor	9.76	11.82	0.00061842	0.016
271	RH_DorsAttn_Post_1	Dorsal Attention	6.13	11.82	0.00062029	0.016
10	LH_Vis_10	Visual	6.53	11.77	0.00063496	0.016

157	LH_Default_Temp_9	Default	7.52	11.73	0.00064960	0.016
154	LH_Default_Temp_6	Default	7.50	11.72	0.00065475	0.016
259	RH_SomMot_29	Somatomotor	8.53	11.68	0.00066864	0.016
260	RH_SomMot_30	Somatomotor	8.25	11.58	0.00070421	0.016
21	LH_Vis_21	Visual	10.61	11.56	0.00070925	0.016
359	RH_Cont_Cing_2	Frontalparietal	8.75	11.54	0.00071988	0.016
124	LH_Limbic_TempPole_6	Limbic	8.67	11.48	0.00074051	0.016
251	RH_SomMot_21	Somatomotor	8.51	11.43	0.00076202	0.016
393	RH_Default_PCC_2	Default	7.79	11.33	0.00080211	0.015
106	LH_SalVentAttn_PFCl_1	Ventral Attention	7.44	11.23	0.00084695	0.015
370	RH_Default_Temp_4	Default	7.45	11.23	0.00084832	0.015
162	LH_Default_Temp_14	Default	7.81	11.19	0.00086544	0.015
147	LH_Cont_Cing_2	Frontalparietal	7.35	11.16	0.00087936	0.015
177	LH_Default_PFC_12	Default	8.23	11.11	0.00090427	0.015
216	RH_Vis_16	Visual	8.27	11.06	0.00092752	0.015
420	Cerebellum_Vermis_VI	Cerebellum	7.23	11.05	0.00093350	0.015
426	Cerebellum_Vermis_CrusII	Cerebellum	6.70	10.97	0.00097332	0.015
109	LH_SalVentAttn_Med_3	Ventral Attention	7.59	10.97	0.00097433	0.015
185	LH_Default_PFC_20	Default	6.63	10.93	0.00099334	0.015
310	RH_SalVentAttn_PFCl_1	Ventral Attention	8.25	10.88	0.00102209	0.015
341	RH_Cont_PFCl_1	Frontalparietal	9.54	10.81	0.00105714	0.015
375	RH_Default_PFCv_1	Default	7.33	10.79	0.00106956	0.015
431	Left_Cerebellum_VIIIa	Cerebellum	5.71	10.65	0.00115117	0.015
7	LH_Vis_7	Visual	6.90	10.64	0.00116186	0.015
435	Cerebellum_Vermis_VIIIb	Cerebellum	9.14	10.55	0.00121767	0.014
28	LH_Vis_28	Visual	9.87	10.37	0.00134024	0.014
193	LH_Default_PCC_4	Default	9.04	10.32	0.00137548	0.014
119	LH_Limbic_TempPole_1	Limbic	7.45	10.27	0.00141287	0.014
178	LH_Default_PFC_13	Default	8.47	10.21	0.00145520	0.014
263	RH_SomMot_33	Somatomotor	10.60	10.20	0.00146636	0.014
302	RH_SalVentAttn_FrOper_1	Ventral Attention	5.13	10.16	0.00149788	0.014
326	RH_Limbic_TempPole_2	Limbic	5.49	10.01	0.00162081	0.014
323	RH_Limbic_OFC_5	Limbic	7.58	10.01	0.00162120	0.014
47	LH_SomMot_16	Somatomotor	7.92	10.00	0.00163431	0.014
406	Left_Amygdala	Subcortical	6.76	9.93	0.00169439	0.014
136	LH_Cont_PFCl_3	Frontalparietal	8.68	9.91	0.00170726	0.014
371	RH_Default_Temp_5	Default	6.98	9.90	0.00171701	0.014
156	LH_Default_Temp_8	Default	6.53	9.84	0.00177433	0.013
239	RH_SomMot_9	Somatomotor	8.04	9.83	0.00178897	0.013
364	RH_Default_Par_3	Default	6.00	9.81	0.00180503	0.013
140	LH_Cont_PFCl_7	Frontalparietal	6.75	9.58	0.00204846	0.013
5	LH_Vis_5	Visual	4.70	9.56	0.00205999	0.013
212	RH_Vis_12	Visual	6.58	9.56	0.00206437	0.013
129	LH_Cont_Par_3	Frontalparietal	6.29	9.50	0.00213073	0.013
419	Left_Cerebellum_VI	Cerebellum	3.56	9.48	0.00215162	0.013
165	LH_Default_Temp_17	Default	6.99	9.46	0.00218327	0.013
208	RH_Vis_8	Visual	8.61	9.38	0.00227512	0.013
125	LH_Limbic_TempPole_7	Limbic	7.02	9.35	0.00230880	0.013
1	LH_Vis_1	Visual	5.89	9.32	0.00235490	0.013
192	LH_Default_PCC_3	Default	8.36	9.25	0.00243765	0.013

69	LH_DorsAttn_Post_1	Dorsal Attention	6.83	9.18	0.00253685	0.013
150	LH_Default_Temp_2	Default	7.08	9.17	0.00255446	0.013
146	LH_Cont_Cing_1	Frontalparietal	7.46	9.13	0.00259899	0.013
322	RH_Limbic_OFC_4	Limbic	9.25	8.99	0.00280064	0.012
280	RH_DorsAttn_Post_10	Dorsal Attention	5.31	8.99	0.00280730	0.012
416	Right_Cerebellum_I_IV	Cerebellum	5.60	8.96	0.00285538	0.012
256	RH_SomMot_26	Somatomotor	8.24	8.95	0.00287670	0.012
298	RH_SalVentAttn_TempOccPar_5	Ventral Attention	7.27	8.90	0.00295590	0.012
437	Left_Cerebellum_IX	Cerebellum	7.07	8.78	0.00314694	0.012
73	LH_DorsAttn_Post_5	Dorsal Attention	11.33	8.76	0.00318176	0.012
230	RH_Vis_30	Visual	8.60	8.68	0.00331333	0.012
97	LH_SalVentAttn_FrOper_1	Ventral Attention	6.57	8.68	0.00331711	0.012
312	RH_SalVentAttn_Med_2	Ventral Attention	7.02	8.68	0.00332596	0.012
254	RH_SomMot_24	Somatomotor	7.01	8.65	0.00338165	0.012
255	RH_SomMot_25	Somatomotor	8.48	8.64	0.00339299	0.012
320	RH_Limbic_OFC_2	Limbic	6.06	8.57	0.00351710	0.012
96	LH_SalVentAttn_TempOcc_1	Ventral Attention	8.77	8.57	0.00352021	0.012
369	RH_Default_Temp_3	Default	6.51	8.56	0.00354921	0.012
226	RH_Vis_26	Visual	9.16	8.54	0.00357699	0.012
222	RH_Vis_22	Visual	7.89	8.42	0.00383214	0.012
330	RH_Limbic_TempPole_6	Limbic	5.83	8.39	0.00389431	0.011
176	LH_Default_PFC_11	Default	8.43	8.38	0.00391712	0.011
241	RH_SomMot_11	Somatomotor	9.09	8.36	0.00394366	0.011
303	RH_SalVentAttn_FrOper_2	Ventral Attention	5.78	8.33	0.00400528	0.011
316	RH_SalVentAttn_Med_6	Ventral Attention	6.16	8.30	0.00408073	0.011
218	RH_Vis_18	Visual	8.36	8.04	0.00469285	0.011
421	Right_Cerebellum_VI	Cerebellum	3.07	8.00	0.00481863	0.011
352	RH_Cont_PFCl_12	Frontalparietal	4.23	7.95	0.00493223	0.011
110	LH_SalVentAttn_Med_4	Ventral Attention	5.91	7.94	0.00496195	0.011
229	RH_Vis_29	Visual	8.54	7.91	0.00503703	0.011
210	RH_Vis_10	Visual	7.81	7.89	0.00511379	0.011
438	Cerebellum_Vermis_IX	Cerebellum	10.04	7.85	0.00520547	0.011
321	RH_Limbic_OFC_3	Limbic	6.78	7.82	0.00529063	0.011
328	RH_Limbic_TempPole_4	Limbic	7.11	7.82	0.00529831	0.011
362	RH_Default_Par_1	Default	7.02	7.76	0.00547510	0.011
127	LH_Cont_Par_1	Frontalparietal	8.29	7.75	0.00551148	0.011
422	Left_Cerebellum_CrusI	Cerebellum	3.70	7.75	0.00551501	0.011
139	LH_Cont_PFCl_6	Frontalparietal	7.43	7.74	0.00554995	0.011
9	LH_Vis_9	Visual	8.04	7.70	0.00566755	0.011
418	Right_Cerebellum_V	Cerebellum	4.81	7.69	0.00570057	0.011
377	RH_Default_PFCv_3	Default	6.73	7.62	0.00592941	0.010
103	LH_SalVentAttn_FrOper_7	Ventral Attention	6.36	7.59	0.00601013	0.010
151	LH_Default_Temp_3	Default	6.19	7.58	0.00603841	0.010
396	RH_Default_PCC_5	Default	8.56	7.58	0.00606077	0.010
104	LH_SalVentAttn_FrOper_8	Ventral Attention	6.66	7.57	0.00607207	0.010
30	LH_Vis_30	Visual	8.30	7.57	0.00608890	0.010
158	LH_Default_Temp_10	Default	7.06	7.54	0.00618224	0.010
209	RH_Vis_9	Visual	6.36	7.52	0.00625441	0.010
327	RH_Limbic_TempPole_3	Limbic	6.30	7.51	0.00627594	0.010
376	RH_Default_PFCv_2	Default	7.40	7.46	0.00645237	0.010

211	RH_Vis_11	Visual	6.31	7.43	0.00655251	0.010
91	LH_DorsAttn_PrCv_2	Dorsal Attention	6.29	7.40	0.00669715	0.010
51	LH_SomMot_20	Somatomotor	8.33	7.34	0.00692223	0.010
339	RH_Cont_Temp_2	Frontalparietal	6.99	7.32	0.00697182	0.010
372	RH_Default_Temp_6	Default	7.08	7.29	0.00709768	0.010
436	Right_Cerebellum_VIIIb	Cerebellum	5.44	7.29	0.00710856	0.010
347	RH_Cont_PFCl_7	Frontalparietal	5.66	7.23	0.00733674	0.010
32	LH_SomMot_1	Somatomotor	5.76	7.23	0.00734748	0.010
383	RH_Default_PFCm_5	Default	7.86	7.19	0.00751805	0.010
11	LH_Vis_11	Visual	7.79	7.13	0.00775292	0.010
182	LH_Default_PFC_17	Default	5.37	7.11	0.00785700	0.010
410	Right_Putamen	Subcortical	3.22	7.00	0.00831832	0.010
365	RH_Default_Par_4	Default	6.29	6.94	0.00860377	0.010
424	Right_Cerebellum_CrusI	Cerebellum	3.02	6.94	0.00860425	0.010
153	LH_Default_Temp_5	Default	5.26	6.93	0.00867867	0.010
114	LH_Limbic_OFC_1	Limbic	6.09	6.92	0.00871918	0.010
272	RH_DorsAttn_Post_2	Dorsal Attention	6.26	6.83	0.00914502	0.009
380	RH_Default_PFCm_2	Default	8.45	6.82	0.00919397	0.009
381	RH_Default_PFCm_3	Default	7.97	6.79	0.00935031	0.009
108	LH_SalVentAttn_Med_2	Ventral Attention	5.61	6.79	0.00937656	0.009
77	LH_DorsAttn_Post_9	Dorsal Attention	6.18	6.76	0.00952942	0.009
204	RH_Vis_4	Visual	7.41	6.71	0.00976861	0.009
55	LH_SomMot_24	Somatomotor	7.54	6.67	0.00997643	0.009
86	LH_DorsAttn_FEF_1	Dorsal Attention	6.06	6.65	0.01012292	0.009
27	LH_Vis_27	Visual	8.52	6.64	0.01018293	0.009
296	RH_SalVentAttn_TempOccPar_3	Ventral Attention	6.60	6.63	0.01021515	0.009
194	LH_Default_PCC_5	Default	8.59	6.53	0.01078631	0.009
180	LH_Default_PFC_15	Default	5.78	6.53	0.01080973	0.009
90	LH_DorsAttn_PrCv_1	Dorsal Attention	7.10	6.52	0.01089206	0.009
117	LH_Limbic_OFC_4	Limbic	6.96	6.50	0.01098120	0.009
135	LH_Cont_PFCl_2	Frontalparietal	6.61	6.49	0.01102505	0.009
195	LH_Default_PCC_6	Default	7.61	6.45	0.01129197	0.009
428	Left_Cerebellum_VIIb	Cerebellum	4.50	6.45	0.01131916	0.009
388	RH_Default_PFCm_10	Default	6.45	6.38	0.01176825	0.009
173	LH_Default_PFC_8	Default	8.30	6.38	0.01177678	0.009
243	RH_SomMot_13	Somatomotor	8.65	6.37	0.01183814	0.009
132	LH_Cont_Par_6	Frontalparietal	5.87	6.31	0.01225596	0.009
386	RH_Default_PFCm_8	Default	5.95	6.30	0.01231580	0.009
57	LH_SomMot_26	Somatomotor	8.62	6.27	0.01251335	0.009
181	LH_Default_PFC_16	Default	5.37	6.25	0.01264559	0.009
95	LH_SalVentAttn_ParOper_4	Ventral Attention	6.26	6.20	0.01302184	0.009
98	LH_SalVentAttn_FrOper_2	Ventral Attention	5.77	6.19	0.01306455	0.009
49	LH_SomMot_18	Somatomotor	6.15	6.19	0.01307612	0.009
133	LH_Cont_Temp_1	Frontalparietal	5.79	6.12	0.01362512	0.008
279	RH_DorsAttn_Post_9	Dorsal Attention	6.43	6.11	0.01370753	0.008
442	Right_Cerebellum_X	Cerebellum	4.94	6.07	0.01399444	0.008
290	RH_DorsAttn_FEF_1	Dorsal Attention	5.90	6.06	0.01405834	0.008
379	RH_Default_PFCm_1	Default	7.66	6.05	0.01417376	0.008
394	RH_Default_PCC_3	Default	9.14	6.04	0.01420442	0.008
337	RH_Cont_Par_6	Frontalparietal	4.18	6.01	0.01442938	0.008

413	Right_Amygdala	Subcortical	5.40	6.01	0.01446565	0.008
281	RH_DorsAttn_Post_11	Dorsal Attention	5.39	6.01	0.01447834	0.008
172	LH_Default_PFC_7	Default	7.52	5.99	0.01458838	0.008
247	RH_SomMot_17	Somatomotor	7.15	5.99	0.01459134	0.008
56	LH_SomMot_25	Somatomotor	7.94	5.97	0.01477742	0.008
354	RH_Cont_PFCl_14	Frontalparietal	4.52	5.95	0.01496055	0.008
175	LH_Default_PFC_10	Default	5.66	5.95	0.01496109	0.008
297	RH_SalVentAttn_TempOccPar_4	Ventral Attention	7.63	5.93	0.01516002	0.008
357	RH_Cont_pCun_2	Frontalparietal	7.39	5.91	0.01528102	0.008
19	LH_Vis_19	Visual	9.79	5.90	0.01534916	0.008
161	LH_Default_Temp_13	Default	9.22	5.89	0.01544963	0.008
116	LH_Limbic_OFC_3	Limbic	5.83	5.89	0.01546695	0.008
334	RH_Cont_Par_3	Frontalparietal	5.08	5.89	0.01551404	0.008
142	LH_Cont_PFCl_9	Frontalparietal	6.36	5.85	0.01581845	0.008
213	RH_Vis_13	Visual	8.87	5.82	0.01613403	0.008
227	RH_Vis_27	Visual	10.13	5.74	0.01679978	0.008
122	LH_Limbic_TempPole_4	Limbic	5.67	5.70	0.01718319	0.008
31	LH_Vis_31	Visual	7.98	5.65	0.01774181	0.008
145	LH_Cont_pCun_2	Frontalparietal	8.89	5.61	0.01816905	0.008
249	RH_SomMot_19	Somatomotor	5.90	5.49	0.01942107	0.008
228	RH_Vis_28	Visual	8.00	5.45	0.01984666	0.008
12	LH_Vis_12	Visual	9.18	5.43	0.02001782	0.007
382	RH_Default_PFCm_4	Default	6.47	5.40	0.02039106	0.007
14	LH_Vis_14	Visual	10.62	5.40	0.02047080	0.007
287	RH_DorsAttn_Post_17	Dorsal Attention	6.55	5.39	0.02048348	0.007
29	LH_Vis_29	Visual	7.65	5.37	0.02079227	0.007
268	RH_SomMot_38	Somatomotor	14.39	5.37	0.02081737	0.007
264	RH_SomMot_34	Somatomotor	8.48	5.30	0.02159485	0.007
348	RH_Cont_PFCl_8	Frontalparietal	6.08	5.29	0.02168126	0.007
54	LH_SomMot_23	Somatomotor	7.23	5.24	0.02230160	0.007
74	LH_DorsAttn_Post_6	Dorsal Attention	4.79	5.09	0.02440073	0.007
314	RH_SalVentAttn_Med_4	Ventral Attention	8.72	5.05	0.02495304	0.007
353	RH_Cont_PFCl_13	Frontalparietal	4.20	4.99	0.02584284	0.007
215	RH_Vis_15	Visual	8.90	4.97	0.02608384	0.007
273	RH_DorsAttn_Post_3	Dorsal Attention	6.45	4.95	0.02639871	0.007
390	RH_Default_PFCm_12	Default	5.69	4.94	0.02656530	0.007
361	RH_Cont_PFCmp_2	Frontalparietal	6.41	4.88	0.02756086	0.007
300	RH_SalVentAttn_TempOccPar_7	Ventral Attention	5.58	4.87	0.02760076	0.007
278	RH_DorsAttn_Post_8	Dorsal Attention	4.17	4.73	0.02991802	0.007
366	RH_Default_Par_5	Default	5.06	4.61	0.03218247	0.006
355	RH_Cont_PFCl_15	Frontalparietal	5.30	4.55	0.03318803	0.006
385	RH_Default_PFCm_7	Default	4.82	4.54	0.03346210	0.006
88	LH_DorsAttn_FEF_3	Dorsal Attention	6.09	4.54	0.03347041	0.006
168	LH_Default_PFC_3	Default	7.62	4.51	0.03407884	0.006
332	RH_Cont_Par_1	Frontalparietal	5.48	4.50	0.03417222	0.006
75	LH_DorsAttn_Post_7	Dorsal Attention	7.55	4.40	0.03628278	0.006
115	LH_Limbic_OFC_2	Limbic	6.14	4.34	0.03762193	0.006
76	LH_DorsAttn_Post_8	Dorsal Attention	5.27	4.30	0.03836428	0.006
179	LH_Default_PFC_14	Default	7.46	4.27	0.03921011	0.006
53	LH_SomMot_22	Somatomotor	6.82	4.27	0.03922059	0.006

402	Left_Caudate	Subcortical	4.95	4.24	0.03979177	0.006
105	LH_SalVentAttn_FrOper_9	Ventral Attention	5.52	4.21	0.04045502	0.006
329	RH_Limbic_TempPole_5	Limbic	4.92	4.06	0.04437770	0.006
199	LH_Default_PCC_10	Default	5.91	3.98	0.04642711	0.005
25	LH_Vis_25	Visual	8.55	3.97	0.04681240	0.005
414	Right_Accumbens	Subcortical	5.11	3.96	0.04709814	0.005
282	RH_DorsAttn_Post_12	Dorsal Attention	5.47	3.94	0.04756540	0.005
131	LH_Cont_Par_5	Frontalparietal	4.51	3.82	0.05111876	0.005
169	LH_Default_PFC_4	Default	7.39	3.71	0.05457219	0.005
417	Left_Cerebellum_V	Cerebellum	4.31	3.67	0.05564456	0.005
315	RH_SalVentAttn_Med_5	Ventral Attention	5.71	3.63	0.05729925	0.005
118	LH_Limbic_OFC_5	Limbic	6.97	3.54	0.06020844	0.005
24	LH_Vis_24	Visual	8.66	3.38	0.06624795	0.005
286	RH_DorsAttn_Post_16	Dorsal Attention	4.41	3.38	0.06646865	0.005
384	RH_Default_PFCm_6	Default	6.64	3.38	0.06647657	0.005
144	LH_Cont_pCun_1	Frontalparietal	5.75	3.32	0.06889596	0.005
335	RH_Cont_Par_4	Frontalparietal	3.14	3.28	0.07034823	0.005
221	RH_Vis_21	Visual	10.40	3.18	0.07494409	0.004
128	LH_Cont_Par_2	Frontalparietal	4.72	3.17	0.07558492	0.004
284	RH_DorsAttn_Post_14	Dorsal Attention	3.50	3.13	0.07707240	0.004
270	RH_SomMot_40	Somatomotor	7.95	3.13	0.07721818	0.004
427	Right_Cerebellum_CrusII	Cerebellum	2.14	3.09	0.07937307	0.004
299	RH_SalVentAttn_TempOccPar_6	Ventral Attention	3.78	3.00	0.08371726	0.004
415	Left_Cerebellum_I_IV	Cerebellum	4.82	2.94	0.08692044	0.004
291	RH_DorsAttn_FEF_2	Dorsal Attention	5.24	2.90	0.08882962	0.004
283	RH_DorsAttn_Post_13	Dorsal Attention	4.61	2.87	0.09089587	0.004
225	RH_Vis_25	Visual	8.50	2.84	0.09215983	0.004
81	LH_DorsAttn_Post_13	Dorsal Attention	6.50	2.83	0.09302948	0.004
269	RH_SomMot_39	Somatomotor	6.26	2.81	0.09419735	0.004
325	RH_Limbic_TempPole_1	Limbic	4.47	2.78	0.09603052	0.004
79	LH_DorsAttn_Post_11	Dorsal Attention	4.60	2.73	0.09893247	0.004
59	LH_SomMot_28	Somatomotor	7.39	2.72	0.09962717	0.004
433	Right_Cerebellum_VIIIa	Cerebellum	3.11	2.57	0.10938604	0.004
83	LH_DorsAttn_Post_15	Dorsal Attention	4.35	2.48	0.11549906	0.003
223	RH_Vis_23	Visual	8.87	2.41	0.12065801	0.003
87	LH_DorsAttn_FEF_2	Dorsal Attention	4.35	2.41	0.12105981	0.003
187	LH_Default_PFC_22	Default	4.10	2.39	0.12244266	0.003
266	RH_SomMot_36	Somatomotor	4.97	2.27	0.13202715	0.003
274	RH_DorsAttn_Post_4	Dorsal Attention	5.90	2.24	0.13484051	0.003
338	RH_Cont_Temp_1	Frontalparietal	3.21	2.22	0.13626070	0.003
336	RH_Cont_Par_5	Frontalparietal	2.55	2.15	0.14339375	0.003
164	LH_Default_Temp_16	Default	6.70	2.05	0.15241516	0.003
130	LH_Cont_Par_4	Frontalparietal	4.93	2.02	0.15534330	0.003
403	Left_Putamen	Subcortical	1.57	2.00	0.15723575	0.003
184	LH_Default_PFC_19	Default	3.88	1.97	0.16090734	0.003
253	RH_SomMot_23	Somatomotor	3.63	1.96	0.16179609	0.003
148	LH_Cont_PFCmp_1	Frontalparietal	5.54	1.86	0.17249045	0.003
409	Right_Caudate	Subcortical	4.48	1.83	0.17603056	0.003
425	Left_Cerebellum_CrusII	Cerebellum	2.73	1.79	0.18147998	0.002
276	RH_DorsAttn_Post_6	Dorsal Attention	2.69	1.77	0.18369566	0.002

430	Right_Cerebellum_VIIb	Cerebellum	2.53	1.72	0.19027705	0.002
324	RH_Limbic_OFC_6	Limbic	5.28	1.71	0.19076219	0.002
60	LH_SomMot_29	Somatomotor	4.64	1.69	0.19406860	0.002
333	RH_Cont_Par_2	Frontalparietal	2.19	1.66	0.19778811	0.002
356	RH_Cont_pCun_1	Frontalparietal	5.36	1.64	0.20062463	0.002
285	RH_DorsAttn_Post_15	Dorsal Attention	4.74	1.58	0.20854470	0.002
112	LH_SalVentAttn_Med_6	Ventral Attention	4.27	1.46	0.22734490	0.002
261	RH_SomMot_31	Somatomotor	4.26	1.40	0.23679291	0.002
404	Left_Pallidum	Subcortical	-3.86	1.39	0.23927538	0.002
52	LH_SomMot_21	Somatomotor	3.12	1.36	0.24332625	0.002
407	Left_Accumbens	Subcortical	3.33	1.35	0.24530748	0.002
63	LH_SomMot_32	Somatomotor	5.56	1.31	0.25220902	0.002
317	RH_SalVentAttn_Med_7	Ventral Attention	3.85	1.04	0.30821858	0.001
288	RH_DorsAttn_Post_18	Dorsal Attention	2.84	1.01	0.31529886	0.001
389	RH_Default_PFCm_11	Default	2.93	0.94	0.33258519	0.001
84	LH_DorsAttn_Post_16	Dorsal Attention	1.97	0.93	0.33448338	0.001
186	LH_Default_PFC_21	Default	2.11	0.74	0.38853627	0.001
189	LH_Default_PFC_24	Default	2.58	0.73	0.39276250	0.001
292	RH_DorsAttn_FEF_3	Dorsal Attention	2.61	0.67	0.41378713	0.001
78	LH_DorsAttn_Post_10	Dorsal Attention	3.41	0.59	0.44301137	0.001
64	LH_SomMot_33	Somatomotor	5.51	0.59	0.44435315	0.001
387	RH_Default_PFCm_9	Default	3.96	0.59	0.44454146	0.001
67	LH_SomMot_36	Somatomotor	2.57	0.58	0.44772459	0.001
62	LH_SomMot_31	Somatomotor	4.77	0.48	0.48778903	0.001
82	LH_DorsAttn_Post_14	Dorsal Attention	2.48	0.44	0.50860386	0.001
188	LH_Default_PFC_23	Default	1.62	0.44	0.50871875	0.001
65	LH_SomMot_34	Somatomotor	-1.09	0.42	0.51826159	0.001
85	LH_DorsAttn_Post_17	Dorsal Attention	1.28	0.37	0.54066748	0.001
89	LH_DorsAttn_FEF_4	Dorsal Attention	3.53	0.36	0.55014386	0.000
262	RH_SomMot_32	Somatomotor	2.68	0.35	0.55149792	0.000
66	LH_SomMot_35	Somatomotor	4.81	0.32	0.56929824	0.000
80	LH_DorsAttn_Post_12	Dorsal Attention	0.81	0.30	0.58141241	0.000
183	LH_Default_PFC_18	Default	1.11	0.20	0.65499368	0.000
391	RH_Default_PFCm_13	Default	2.16	0.18	0.67379195	0.000
277	RH_DorsAttn_Post_7	Dorsal Attention	3.17	0.17	0.67690082	0.000
68	LH_SomMot_37	Somatomotor	4.77	0.15	0.69693912	0.000
318	RH_SalVentAttn_Med_8	Ventral Attention	0.87	0.09	0.76112717	0.000
267	RH_SomMot_37	Somatomotor	1.50	0.08	0.77359714	0.000
411	Right_Pallidum	Subcortical	-0.97	0.08	0.77910264	0.000
58	LH_SomMot_27	Somatomotor	2.06	0.07	0.78524898	0.000
289	RH_DorsAttn_Post_19	Dorsal Attention	1.30	0.06	0.80503706	0.000
258	RH_SomMot_28	Somatomotor	0.73	0.02	0.90170967	0.000
265	RH_SomMot_35	Somatomotor	0.96	0.02	0.90188428	0.000
61	LH_SomMot_30	Somatomotor	2.78	0.01	0.92203666	0.000
257	RH_SomMot_27	Somatomotor	0.32	0.01	0.92693173	0.000
113	LH_SalVentAttn_Med_7	Ventral Attention	0.23	0.00	0.95044972	0.000

Supplementary Table 3. The potential brain age mediators of the relationships between CSVD and global cognitive performance (MMSE)

ROI Index	Region	Network	*Path a*				*Path b*				*Path ab*				*Path c'*				*Path c*				% mediation
			ß	SE	z	p	ß	SE	z	p	ß	SE	z	p	ß	SE	z	p	ß	SE	z	p	
401	Left Thalamus	Subcortical	3.94	0.73	3.70	**0.0002**	-0.03	0.01	-2.23	**0.02548**	-0.11	0.05	-2.10	**0.0358**	-0.67	0.34	-1.96	0.05055	-0.78	0.34	-2.30	**0.0216**	14%
405	Left Hippocampus	Subcortical	5.86	1.15	3.71	**0.0002**	-0.02	0.01	-2.22	**0.02645**	-0.10	0.05	-2.10	**0.0360**	-0.68	0.34	-2.01	**0.04472**	-0.79	0.34	-2.32	**0.0201**	13%
231	RH_SomMot_1	Somatomotor	5.98	1.18	3.69	**0.0002**	-0.02	0.01	-2.09	**0.03629**	-0.09	0.05	-1.96	**0.0497**	-0.69	0.34	-2.02	**0.04331**	-0.78	0.34	-2.29	**0.0222**	12%
248	RH_SomMot_18	Somatomotor	6.72	1.41	3.68	**0.0002**	-0.01	0.01	-2.11	**0.03521**	-0.09	0.05	-1.93	0.0535	-0.69	0.34	-2.03	**0.04250**	-0.78	0.34	-2.28	**0.0227**	12%
367	RH_Default_Temp_1	Default	7.51	1.50	3.73	**0.0002**	-0.01	0.01	-1.79	0.07287	-0.10	0.06	-1.70	0.0900	-0.69	0.34	-2.02	**0.04299**	-0.79	0.34	-2.35	**0.0187**	12%
246	RH_SomMot_16	Somatomotor	7.85	1.43	3.71	**0.0002**	-0.01	0.01	-1.81	0.07015	-0.08	0.05	-1.68	0.0921	-0.70	0.34	-1.97	**0.04841**	-0.78	0.34	-2.23	**0.0261**	11%
242	RH_SomMot_12	Somatomotor	7.88	1.54	3.62	**0.0003**	-0.01	0.01	-1.74	0.08201	-0.08	0.05	-1.59	0.1115	-0.70	0.34	-2.02	**0.04377**	-0.78	0.34	-2.29	**0.0218**	10%
102	LH_SalVentAttn_FrOper_6	Ventral Attention	7.09	1.58	3.68	**0.0002**	-0.01	0.01	-1.68	0.09214	-0.08	0.06	-1.55	0.1213	-0.70	0.34	-2.03	**0.04220**	-0.78	0.34	-2.27	**0.0232**	11%
235	RH_SomMot_5	Somatomotor	6.56	1.49	4.14	**0.0000**	-0.01	0.01	-1.64	0.10191	-0.08	0.05	-1.51	0.1312	-0.71	0.34	-2.07	**0.03858**	-0.79	0.34	-2.27	**0.0229**	10%
245	RH_SomMot_15	Somatomotor	10.08	1.88	3.72	**0.0002**	-0.01	0.01	-1.58	0.11399	-0.09	0.06	-1.48	0.1400	-0.69	0.34	-1.94	0.05215	-0.78	0.34	-2.24	**0.0251**	12%
17	LH_Vis_17	Visual	10.43	2.23	3.76	**0.0002**	-0.01	0.00	-1.40	0.16267	-0.06	0.05	-1.30	0.1939	-0.73	0.34	-2.24	**0.02529**	-0.79	0.34	-2.37	**0.0179**	8%
306	RH_SalVentAttn_FrOper_5	Ventral Attention	8.49	1.38	3.68	**0.0002**	-0.01	0.01	-1.30	0.19431	-0.09	0.07	-1.23	0.2188	-0.69	0.35	-1.92	0.05462	-0.78	0.34	-2.25	**0.0242**	12%
351	RH_Cont_PFCl_11	Frontoparietal	8.66	1.78	3.67	**0.0002**	0.01	0.01	1.00	0.31890	0.05	0.05	1.08	0.2798	-0.84	0.34	-2.47	**0.01334**	-0.79	0.33	-2.35	**0.0189**	7%
307	RH_SalVentAttn_FrOper_6	Ventral Attention	6.64	1.37	3.69	**0.0002**	-0.01	0.01	-1.16	0.24436	-0.05	0.05	-1.07	0.2837	-0.73	0.35	-2.08	**0.03727**	-0.78	0.34	-2.30	**0.0215**	7%
214	RH_Vis_14	Visual	7.54	1.41	3.73	**0.0002**	-0.01	0.01	-1.12	0.26261	-0.06	0.06	-1.05	0.2931	-0.72	0.34	-2.07	**0.03810**	-0.78	0.34	-2.28	**0.0228**	8%
220	RH_Vis_20	Visual	9.11	1.57	3.70	**0.0002**	0.01	0.01	0.96	0.33508	0.06	0.06	1.04	0.2997	-0.84	0.35	-2.51	**0.01222**	-0.79	0.34	-2.34	**0.0193**	7%
305	RH_SalVentAttn_FrOper_4	Ventral Attention	6.07	1.16	3.72	**0.0002**	-0.01	0.01	-1.13	0.25766	-0.05	0.05	-1.03	0.3010	-0.73	0.34	-2.14	**0.03263**	-0.79	0.34	-2.30	**0.0214**	6%
238	RH_SomMot_8	Somatomotor	8.17	1.75	3.67	**0.0002**	-0.01	0.01	-1.12	0.26087	-0.06	0.06	-1.02	0.3084	-0.72	0.33	-2.08	**0.03759**	-0.78	0.33	-2.25	**0.0247**	8%
408	Right Thalamus	Subcortical	3.60	0.76	3.72	**0.0002**	-0.01	0.01	-1.10	0.27231	-0.05	0.05	-0.98	0.3253	-0.73	0.34	-2.19	**0.02881**	-0.79	0.34	-2.34	**0.0191**	7%
397	RH_Default_PCC_6	Default	9.62	1.87	3.75	**0.0002**	-0.01	0.01	-1.01	0.31293	-0.06	0.06	-0.94	0.3486	-0.72	0.34	-2.13	**0.03308**	-0.78	0.34	-2.36	**0.0181**	7%
202	RH_Vis_2	Visual	6.42	1.39	3.66	**0.0003**	-0.01	0.01	-0.92	0.35748	-0.04	0.05	-0.84	0.4009	-0.75	0.34	-2.22	**0.02615**	-0.79	0.34	-2.33	**0.0198**	5%
23	LH_Vis_23	Visual	8.37	2.02	3.78	**0.0002**	-0.01	0.01	-0.96	0.33808	-0.04	0.05	-0.83	0.4052	-0.74	0.33	-2.24	**0.02493**	-0.79	0.34	-2.37	**0.0177**	6%
434	Left_Cerebellum_VIIIb	Cerebellum	7.06	1.60	3.61	**0.0003**	0.01	0.01	0.73	0.46727	0.04	0.05	0.82	0.4112	-0.82	0.34	-2.39	**0.01707**	-0.78	0.34	-2.31	**0.0209**	5%
234	RH_SomMot_4	Somatomotor	6.50	1.27	3.67	**0.0002**	-0.01	0.01	-0.89	0.37084	-0.04	0.05	-0.82	0.4116	-0.74	0.34	-2.12	**0.03422**	-0.78	0.34	-2.29	**0.0218**	5%
72	LH_DorsAttn_Post_4	Dorsal Attention	10.32	1.91	3.71	**0.0002**	0.00	0.01	-0.87	0.38385	-0.04	0.05	-0.81	0.4159	-0.74	0.34	-2.20	**0.02766**	-0.78	0.33	-2.29	**0.0218**	6%
217	RH_Vis_17	Visual	6.37	1.51	3.74	**0.0002**	-0.01	0.01	-0.89	0.37345	-0.04	0.05	-0.81	0.4174	-0.74	0.35	-2.09	**0.03702**	-0.78	0.34	-2.26	**0.0240**	6%
429	Cerebellum_Vermis_VIIb	Cerebellum	8.71	1.99	3.80	**0.0001**	-0.01	0.01	-0.86	0.39116	-0.05	0.05	-0.78	0.4364	-0.73	0.34	-2.10	**0.03567**	-0.78	0.34	-2.23	**0.0258**	6%
342	RH_Cont_PFCl_2	Frontoparietal	9.03	1.95	3.70	**0.0002**	0.00	0.01	-0.85	0.39439	-0.04	0.05	-0.77	0.4393	-0.74	0.33	-2.20	**0.02761**	-0.78	0.33	-2.33	**0.0197**	5%
398	RH_Default_PCC_7	Default	6.85	1.56	3.70	**0.0002**	-0.01	0.01	-0.84	0.39990	-0.04	0.05	-0.77	0.4405	-0.75	0.34	-2.18	**0.02914**	-0.79	0.34	-2.30	**0.0217**	5%
155	LH_Default_Temp_7	Default	7.80	1.66	3.70	**0.0002**	0.00	0.01	0.70	0.48408	0.03	0.05	0.77	0.4422	-0.81	0.34	-2.35	**0.01896**	-0.78	0.34	-2.25	**0.0246**	4%
15	LH_Vis_15	Visual	7.79	1.95	3.77	**0.0002**	-0.01	0.01	-0.90	0.36867	-0.04	0.05	-0.77	0.4436	-0.74	0.33	-2.18	**0.02917**	-0.78	0.33	-2.29	**0.0222**	5%
240	RH_SomMot_10	Somatomotor	8.56	1.51	3.72	**0.0002**	0.00	0.01	-0.82	0.41288	-0.04	0.05	-0.76	0.4448	-0.74	0.34	-2.16	**0.03115**	-0.79	0.34	-2.35	**0.0186**	5%
295	RH_SalVentAttn_TempOccPar_2	Ventral Attention	7.58	1.96	3.63	**0.0003**	-0.01	0.01	-0.90	0.36780	-0.04	0.05	-0.75	0.4514	-0.75	0.34	-2.17	**0.03025**	-0.78	0.34	-2.31	**0.0209**	5%
198	LH_Default_PCC_9	Default	7.26	1.63	3.68	**0.0002**	-0.01	0.01	-0.84	0.40251	-0.04	0.05	-0.74	0.4568	-0.74	0.34	-2.18	**0.02952**	-0.78	0.34	-2.25	**0.0243**	5%
159	LH_Default_Temp_11	Default	8.25	1.75	3.68	**0.0002**	0.00	0.00	-0.81	0.41814	-0.03	0.04	-0.73	0.4626	-0.76	0.34	-2.22	**0.02668**	-0.79	0.34	-2.41	**0.0160**	4%
143	LH_Cont_PFCv_1	Frontoparietal	6.16	1.40	3.97	**0.0001**	-0.01	0.01	-0.78	0.43400	-0.04	0.05	-0.72	0.4703	-0.75	0.34	-2.24	**0.02512**	-0.79	0.34	-2.33	**0.0196**	5%
44	LH_SomMot_13	Somatomotor	8.73	1.51	3.71	**0.0002**	0.00	0.01	0.67	0.50550	0.04	0.05	0.71	0.4801	-0.82	0.34	-2.37	**0.01803**	-0.78	0.34	-2.26	**0.0237**	5%
41	LH_SomMot_10	Somatomotor	8.25	1.59	3.68	**0.0002**	0.00	0.01	0.64	0.52389	0.03	0.05	0.70	0.4835	-0.81	0.35	-2.27	**0.02315**	-0.78	0.34	-2.26	**0.0239**	4%
160	LH_Default_Temp_12	Default	10.06	1.95	3.69	**0.0002**	0.00	0.01	-0.77	0.44344	-0.04	0.05	-0.70	0.4871	-0.75	0.34	-2.24	**0.02487**	-0.79	0.34	-2.39	**0.0170**	5%
344	RH_Cont_PFCl_4	Frontoparietal	9.36	1.86	3.69	**0.0002**	0.00	0.00	-0.69	0.49090	-0.03	0.05	-0.65	0.5149	-0.75	0.34	-2.18	**0.02943**	-0.78	0.34	-2.27	**0.0233**	4%
93	LH_SalVentAttn_ParOper_2	Ventral Attention	8.06	1.79	3.70	**0.0002**	0.00	0.01	-0.72	0.47187	-0.03	0.04	-0.65	0.5181	-0.75	0.34	-2.15	**0.03136**	-0.78	0.34	-2.27	**0.0232**	4%
400	RH_Default_PCC_9	Default	8.87	2.12	3.80	**0.0001**	0.00	0.01	-0.73	0.46594	-0.03	0.05	-0.64	0.5198	-0.76	0.35	-2.21	**0.02718**	-0.79	0.35	-2.30	**0.0214**	4%
40	LH_SomMot_9	Somatomotor	8.06	1.67	3.74	**0.0002**	0.00	0.01	-0.67	0.50254	-0.03	0.05	-0.60	0.5474	-0.75	0.34	-2.22	**0.02675**	-0.79	0.33	-2.29	**0.0217**	4%
293	RH_DorsAttn_PrCv_1	Dorsal Attention	7.90	1.53	3.70	**0.0002**	0.00	0.01	-0.65	0.51615	-0.03	0.05	-0.60	0.5511	-0.76	0.34	-2.12	**0.03423**	-0.78	0.34	-2.22	**0.0265**	4%
43	LH_SomMot_12	Somatomotor	7.38	1.51	3.72	**0.0002**	0.00	0.01	0.54	0.58841	0.02	0.05	0.59	0.5582	-0.80	0.34	-2.34	**0.01935**	-0.78	0.34	-2.29	**0.0223**	3%

107	LH_SalVentAttn_Med_1	Ventral Attention	6.80	1.29	3.64	**0.0003**	0.00	0.01	-0.57	0.56744	-0.03	0.05	-0.52	0.6023	-0.76	0.34	-2.20	**0.02754**	-0.79	0.34	-2.30	**0.0215**	4%
439	Right_Cerebellum_IX	Cerebellum	8.21	1.79	3.69	**0.0002**	0.00	0.01	-0.56	0.57603	-0.03	0.06	-0.52	0.6062	-0.75	0.34	-2.21	**0.02700**	-0.78	0.34	-2.38	**0.0174**	4%
207	RH_Vis_7	Visual	6.65	1.49	3.69	**0.0002**	0.00	0.01	0.44	0.65734	0.02	0.05	0.51	0.6114	-0.80	0.34	-2.32	**0.02010**	-0.78	0.34	-2.28	**0.0224**	2%
42	LH_SomMot_11	Somatomotor	8.11	1.67	3.75	**0.0002**	0.00	0.01	0.46	0.64832	0.02	0.05	0.50	0.6163	-0.80	0.34	-2.29	**0.02195**	-0.78	0.34	-2.26	**0.0239**	3%
48	LH_SomMot_17	Somatomotor	7.02	1.40	3.61	**0.0003**	0.00	0.01	0.47	0.63482	0.02	0.04	0.50	0.6181	-0.80	0.34	-2.33	**0.01988**	-0.78	0.34	-2.25	**0.0242**	3%
197	LH_Default_PCC_8	Default	7.86	1.72	3.67	**0.0002**	0.00	0.01	-0.55	0.58333	-0.03	0.05	-0.50	0.6192	-0.76	0.34	-2.18	**0.02924**	-0.79	0.34	-2.25	**0.0241**	3%
38	LH_SomMot_7	Somatomotor	8.33	1.67	3.72	**0.0002**	0.00	0.01	-0.50	0.61574	-0.03	0.05	-0.47	0.6356	-0.76	0.34	-2.22	**0.02648**	-0.79	0.34	-2.35	**0.0190**	3%
26	LH_Vis_26	Visual	7.38	1.56	3.76	**0.0002**	0.00	0.01	-0.50	0.61516	-0.02	0.05	-0.46	0.6481	-0.76	0.34	-2.25	**0.02476**	-0.78	0.34	-2.34	**0.0193**	3%
304	RH_SalVentAttn_FrOper_3	Ventral Attention	5.28	1.12	3.72	**0.0002**	0.00	0.01	-0.48	0.62918	-0.02	0.04	-0.45	0.6512	-0.76	0.34	-2.22	**0.02614**	-0.78	0.34	-2.26	**0.0237**	2%
121	LH_Limbic_TempPole_3	Limbic	6.78	1.70	3.63	**0.0003**	0.00	0.01	-0.51	0.60784	-0.02	0.05	-0.44	0.6595	-0.77	0.34	-2.29	**0.02219**	-0.79	0.34	-2.36	**0.0185**	3%
373	RH_Default_Temp_7	Default	7.48	1.67	3.72	**0.0002**	0.00	0.01	0.40	0.69283	0.02	0.05	0.43	0.6652	-0.80	0.34	-2.32	**0.02020**	-0.79	0.34	-2.33	**0.0197**	2%
441	Cerebellum_Vermis_X	Cerebellum	12.66	3.02	3.72	**0.0002**	0.00	0.00	-0.49	0.62466	-0.03	0.05	-0.42	0.6754	-0.76	0.34	-2.23	**0.02607**	-0.78	0.34	-2.31	**0.0207**	3%
232	RH_SomMot_2	Somatomotor	7.98	1.56	3.74	**0.0002**	0.00	0.01	0.37	0.71181	0.02	0.06	0.41	0.6835	-0.81	0.34	-2.39	**0.01669**	-0.79	0.34	-2.36	**0.0185**	3%
345	RH_Cont_PFCl_5	Frontoparietal	9.05	1.87	3.72	**0.0002**	0.00	0.01	-0.46	0.64236	-0.02	0.05	-0.41	0.6839	-0.76	0.33	-2.25	**0.02456**	-0.78	0.33	-2.32	**0.0202**	3%
36	LH_SomMot_5	Somatomotor	7.66	1.57	3.83	**0.0001**	0.00	0.01	-0.43	0.66478	-0.02	0.05	-0.40	0.6856	-0.76	0.34	-2.28	**0.02267**	-0.79	0.34	-2.38	**0.0172**	3%
70	LH_DorsAttn_Post_2	Dorsal Attention	8.72	1.68	3.77	**0.0002**	0.00	0.01	0.37	0.70835	0.02	0.06	0.40	0.6882	-0.81	0.34	-2.38	**0.01710**	-0.78	0.34	-2.35	**0.0187**	3%
35	LH_SomMot_4	Somatomotor	6.29	1.37	3.75	**0.0002**	0.00	0.01	-0.46	0.64561	-0.02	0.05	-0.40	0.6913	-0.76	0.34	-2.21	**0.02678**	-0.78	0.34	-2.25	**0.0243**	3%
392	RH_Default_PCC_1	Default	7.44	1.49	3.79	**0.0002**	0.00	0.01	0.35	0.72967	0.02	0.05	0.38	0.7023	-0.80	0.34	-2.34	**0.01949**	-0.78	0.33	-2.34	**0.0193**	2%
224	RH_Vis_24	Visual	8.26	1.62	3.75	**0.0002**	0.00	0.01	0.34	0.73467	0.02	0.05	0.37	0.7129	-0.80	0.34	-2.34	**0.01939**	-0.78	0.34	-2.29	**0.0220**	2%
244	RH_SomMot_14	Somatomotor	7.40	1.59	3.67	**0.0002**	0.00	0.01	-0.38	0.70479	-0.01	0.04	-0.36	0.7209	-0.77	0.35	-2.15	**0.03166**	-0.79	0.34	-2.23	**0.0261**	2%
236	RH_SomMot_6	Somatomotor	8.98	1.50	3.61	**0.0003**	0.00	0.01	-0.38	0.70413	-0.02	0.06	-0.36	0.7221	-0.77	0.35	-2.31	**0.02107**	-0.79	0.34	-2.37	**0.0178**	2%
6	LH_Vis_6	Visual	10.62	2.33	3.64	**0.0003**	0.00	0.00	-0.37	0.70775	-0.02	0.05	-0.32	0.7459	-0.77	0.34	-2.22	**0.02633**	-0.79	0.34	-2.34	**0.0195**	2%
22	LH_Vis_22	Visual	8.79	1.47	3.65	**0.0003**	0.00	0.01	-0.30	0.76741	-0.01	0.06	-0.29	0.7720	-0.78	0.34	-2.28	**0.02267**	-0.79	0.34	-2.34	**0.0193**	2%
149	LH_Default_Temp_1	Default	7.13	1.57	3.81	**0.0001**	0.00	0.01	0.25	0.80098	0.01	0.05	0.28	0.7816	-0.80	0.34	-2.39	**0.01682**	-0.79	0.34	-2.32	**0.0204**	2%
71	LH_DorsAttn_Post_3	Dorsal Attention	11.09	2.14	3.67	**0.0002**	0.00	0.01	-0.27	0.78755	-0.01	0.06	-0.26	0.7952	-0.77	0.34	-2.19	**0.02862**	-0.78	0.34	-2.26	**0.0237**	2%
141	LH_Cont_PFCl_8	Frontoparietal	7.51	1.57	3.68	**0.0002**	0.00	0.01	-0.26	0.79447	-0.01	0.05	-0.24	0.8080	-0.78	0.34	-2.24	**0.02516**	-0.79	0.34	-2.31	**0.0207**	1%
395	RH_Default_PCC_4	Default	6.89	1.56	3.74	**0.0002**	0.00	0.01	-0.27	0.79067	-0.01	0.05	-0.24	0.8093	-0.77	0.34	-2.26	**0.02372**	-0.78	0.34	-2.33	**0.0199**	1%
8	LH_Vis_8	Visual	11.80	2.00	3.71	**0.0002**	0.00	0.01	-0.24	0.80943	-0.02	0.06	-0.21	0.8316	-0.77	0.34	-2.31	**0.02100**	-0.79	0.34	-2.31	**0.0208**	2%
191	LH_Default_PCC_2	Default	6.96	1.61	3.78	**0.0002**	0.00	0.01	0.19	0.85258	0.01	0.04	0.21	0.8353	-0.79	0.34	-2.28	**0.02284**	-0.78	0.34	-2.26	**0.0237**	1%
331	RH_Limbic_TempPole_7	Limbic	6.17	1.36	3.78	**0.0002**	0.00	0.01	0.18	0.85452	0.01	0.05	0.21	0.8353	-0.80	0.34	-2.42	**0.01562**	-0.79	0.34	-2.37	**0.0179**	1%
350	RH_Cont_PFCl_10	Frontoparietal	7.42	1.61	3.77	**0.0002**	0.00	0.01	0.19	0.84670	0.01	0.05	0.20	0.8381	-0.79	0.34	-2.29	**0.02226**	-0.78	0.33	-2.29	**0.0222**	1%
196	LH_Default_PCC_7	Default	9.28	2.00	3.67	**0.0002**	0.00	0.00	0.15	0.88013	0.00	0.05	0.16	0.8738	-0.79	0.34	-2.22	**0.02639**	-0.78	0.34	-2.21	**0.0273**	1%
20	LH_Vis_20	Visual	7.52	1.58	3.65	**0.0003**	0.00	0.01	-0.15	0.87953	0.00	0.05	-0.15	0.8835	-0.78	0.34	-2.25	**0.02460**	-0.78	0.33	-2.33	**0.0197**	0%
99	LH_SalVentAttn_FrOper_3	Ventral Attention	7.13	1.60	3.70	**0.0002**	0.00	0.01	-0.15	0.88286	0.00	0.05	-0.13	0.8928	-0.78	0.34	-2.27	**0.02316**	-0.78	0.34	-2.28	**0.0224**	0%
126	LH_Limbic_TempPole_8	Limbic	6.61	1.48	3.77	**0.0002**	0.00	0.01	0.11	0.90869	0.00	0.05	0.13	0.8964	-0.78	0.34	-2.30	**0.02158**	-0.78	0.34	-2.27	**0.0234**	0%
152	LH_Default_Temp_4	Default	7.75	1.66	3.69	**0.0002**	0.00	0.01	0.11	0.91250	0.01	0.04	0.12	0.9061	-0.79	0.34	-2.36	**0.01822**	-0.79	0.34	-2.37	**0.0177**	1%
16	LH_Vis_16	Visual	7.35	1.45	3.66	**0.0003**	0.00	0.01	-0.12	0.90604	-0.01	0.06	-0.11	0.9164	-0.78	0.35	-2.23	**0.02553**	-0.78	0.34	-2.27	**0.0232**	1%
94	LH_SalVentAttn_ParOper_3	Ventral Attention	7.34	1.68	3.73	**0.0002**	0.00	0.01	0.10	0.92348	0.00	0.04	0.10	0.9175	-0.78	0.33	-2.28	**0.02283**	-0.78	0.34	-2.24	**0.0254**	0%
374	RH_Default_Temp_8	Default	7.68	1.60	3.72	**0.0002**	0.00	0.01	0.09	0.92959	0.01	0.05	0.10	0.9196	-0.79	0.34	-2.28	**0.02259**	-0.78	0.34	-2.21	**0.0273**	1%
311	RH_SalVentAttn_Med_1	Ventral Attention	6.38	1.27	3.62	**0.0003**	0.00	0.01	-0.09	0.92747	-0.01	0.04	-0.09	0.9323	-0.77	0.34	-2.25	**0.02474**	-0.78	0.34	-2.28	**0.0224**	1%
33	LH_SomMot_2	Somatomotor	6.69	1.39	3.70	**0.0002**	0.00	0.01	-0.09	0.92684	0.00	0.04	-0.08	0.9332	-0.78	0.35	-2.18	**0.02946**	-0.78	0.34	-2.24	**0.0248**	0%
45	LH_SomMot_14	Somatomotor	6.71	1.55	3.81	**0.0001**	0.00	0.01	0.05	0.95741	0.00	0.04	0.06	0.9510	-0.79	0.34	-2.36	**0.01851**	-0.79	0.34	-2.34	**0.0193**	0%
2	LH_Vis_2	Visual	6.51	1.41	3.63	**0.0003**	0.00	0.01	0.04	0.96781	0.00	0.05	0.05	0.9634	-0.79	0.33	-2.34	**0.01950**	-0.78	0.33	-2.32	**0.0205**	0%
37	LH_SomMot_6	Somatomotor	8.28	1.82	3.67	**0.0002**	0.00	0.01	0.02	0.98040	0.00	0.04	0.02	0.9812	-0.78	0.34	-2.21	**0.02742**	-0.78	0.34	-2.30	**0.0214**	0%

The mediation regression coefficients (ß), standard errors (SE), Z-scores and p-values for path a, b, ab, c and c'.

% mediation indicates the effect calculated from the proportion between path ab and path c

Supplementary Table 4. The potential brain age mediators of the relationships between CSVD and verbal memory (CVVLT)

ROI Index	Region	Network	*Path a*				*Path b*				*Path ab*				*Path c'*				*Path c*				% mediation
			ß	SE	z	p	ß	SE	z	p	ß	SE	z	p	ß	SE	z	p	ß	SE	z	p	
155	LH_Default_Temp_7	Default	7.78	1.66	3.70	**0.0002**	0.01	0.00	2.10	**0.03564**	0.06	0.03	2.27	**0.0234**	-0.50	0.20	-2.43	**0.01492**	-0.43	0.20	-2.20	**0.0280**	14%
311	RH_SalVentAttn_Med_1	Ventral Attention	6.40	1.30	3.63	**0.0003**	0.01	0.00	2.12	**0.03425**	0.06	0.03	2.27	**0.0235**	-0.50	0.20	-2.46	**0.01383**	-0.43	0.20	-2.18	**0.0294**	15%
351	RH_Cont_PFCl_11	Frontoparietal	8.67	1.82	3.66	**0.0003**	0.01	0.00	1.96	0.05005	0.06	0.04	2.09	**0.0366**	-0.50	0.20	-2.55	**0.01071**	-0.43	0.20	-2.24	**0.0253**	15%
191	LH_Default_PCC_2	Default	6.94	1.60	3.73	**0.0002**	-0.01	0.00	-2.12	**0.03418**	-0.07	0.04	-1.96	0.0503	-0.37	0.19	-1.93	0.05360	-0.44	0.19	-2.29	**0.0220**	15%
141	LH_Cont_PFCl_8	Frontoparietal	7.52	1.57	3.70	**0.0002**	0.01	0.00	1.79	0.07348	0.05	0.03	1.90	0.0570	-0.49	0.20	-2.54	**0.01121**	-0.44	0.19	-2.24	**0.0250**	12%
44	LH_SomMot_13	Somatomotor	8.74	1.50	3.74	**0.0002**	0.01	0.00	1.75	0.07975	0.06	0.04	1.83	0.0673	-0.50	0.20	-2.49	**0.01286**	-0.44	0.20	-2.21	**0.0268**	14%
429	Cerebellum_Vermis_VIIb	Cerebellum	8.68	2.00	3.87	**0.0001**	0.01	0.00	1.59	0.11141	0.04	0.03	1.78	0.0758	-0.48	0.20	-2.41	**0.01586**	-0.44	0.20	-2.16	**0.0308**	10%
306	RH_SalVentAttn_FrOper_5	Ventral Attention	8.51	1.37	3.67	**0.0002**	-0.01	0.01	-1.62	0.10466	-0.07	0.04	-1.56	0.1196	-0.36	0.20	-1.82	0.06804	-0.43	0.20	-2.20	**0.0278**	16%
434	Left_Cerebellum_VIIIb	Cerebellum	7.05	1.61	3.65	**0.0003**	0.01	0.00	1.33	0.18282	0.04	0.03	1.47	0.1419	-0.48	0.20	-2.41	**0.01583**	-0.44	0.20	-2.27	**0.0232**	9%
160	LH_Default_Temp_12	Default	10.07	1.98	3.68	**0.0002**	0.00	0.00	1.37	0.16973	0.05	0.04	1.47	0.1425	-0.49	0.20	-2.47	**0.01369**	-0.44	0.20	-2.27	**0.0230**	11%
45	LH_SomMot_14	Somatomotor	6.75	1.55	3.74	**0.0002**	0.01	0.00	1.23	0.21788	0.04	0.03	1.35	0.1776	-0.47	0.20	-2.34	**0.01913**	-0.43	0.20	-2.17	**0.0301**	8%
35	LH_SomMot_4	Somatomotor	6.33	1.37	3.71	**0.0002**	-0.01	0.00	-1.46	0.14436	-0.04	0.03	-1.34	0.1790	-0.39	0.20	-1.96	**0.04993**	-0.44	0.20	-2.25	**0.0243**	10%
43	LH_SomMot_12	Somatomotor	7.34	1.53	3.79	**0.0002**	0.00	0.00	1.10	0.26970	0.03	0.03	1.25	0.2112	-0.47	0.20	-2.40	**0.01659**	-0.44	0.20	-2.23	**0.0254**	7%
236	RH_SomMot_6	Somatomotor	8.98	1.49	3.62	**0.0003**	-0.01	0.00	-1.27	0.20261	-0.05	0.04	-1.22	0.2211	-0.38	0.20	-1.90	0.05683	-0.43	0.20	-2.17	**0.0299**	11%
235	RH_SomMot_5	Somatomotor	6.56	1.48	4.15	**0.0000**	-0.01	0.01	-1.33	0.18515	-0.04	0.03	-1.20	0.2302	-0.39	0.20	-1.95	0.05058	-0.43	0.20	-2.20	**0.0278**	10%
350	RH_Cont_PFCl_10	Frontoparietal	7.44	1.60	3.72	**0.0002**	0.00	0.00	1.11	0.26690	0.03	0.03	1.20	0.2302	-0.47	0.20	-2.31	**0.02103**	-0.43	0.20	-2.17	**0.0298**	8%
198	LH_Default_PCC_9	Default	7.27	1.64	3.67	**0.0002**	0.00	0.00	1.09	0.27675	0.03	0.03	1.20	0.2310	-0.47	0.20	-2.34	**0.01954**	-0.43	0.20	-2.15	**0.0319**	8%
102	LH_SalVentAttn_FrOper_6	Ventral Attention	7.09	1.55	3.69	**0.0002**	-0.01	0.00	-1.31	0.18871	-0.04	0.03	-1.19	0.2349	-0.39	0.20	-2.03	**0.04202**	-0.44	0.20	-2.27	**0.0232**	10%
202	RH_Vis_2	Visual	6.45	1.40	3.61	**0.0003**	-0.01	0.00	-1.26	0.20934	-0.04	0.03	-1.16	0.2451	-0.40	0.20	-2.02	**0.04291**	-0.43	0.19	-2.20	**0.0280**	9%
48	LH_SomMot_17	Somatomotor	7.00	1.42	3.63	**0.0003**	0.00	0.00	1.06	0.28755	0.03	0.03	1.16	0.2461	-0.46	0.20	-2.28	**0.02286**	-0.43	0.20	-2.14	**0.0325**	7%
26	LH_Vis_26	Visual	7.38	1.59	3.76	**0.0002**	0.00	0.00	-1.21	0.22806	-0.04	0.03	-1.10	0.2708	-0.40	0.20	-2.07	**0.03855**	-0.44	0.20	-2.27	**0.0231**	8%
107	LH_SalVentAttn_Med_1	Ventral Attention	6.79	1.28	3.67	**0.0002**	0.00	0.00	0.99	0.32384	0.03	0.03	1.06	0.2874	-0.47	0.20	-2.37	**0.01787**	-0.44	0.20	-2.24	**0.0252**	7%
42	LH_SomMot_11	Somatomotor	8.12	1.67	3.76	**0.0002**	0.00	0.00	0.96	0.33546	0.03	0.03	1.03	0.3022	-0.47	0.20	-2.37	**0.01761**	-0.44	0.20	-2.32	**0.0205**	7%
121	LH_Limbic_TempPole_3	Limbic	6.74	1.68	3.69	**0.0002**	-0.01	0.00	-1.18	0.23891	-0.04	0.03	-1.02	0.3056	-0.40	0.20	-2.01	**0.04413**	-0.43	0.20	-2.26	**0.0240**	9%
231	RH_SomMot_1	Somatomotor	6.00	1.17	3.67	**0.0002**	-0.01	0.01	-1.10	0.26941	-0.03	0.03	-0.99	0.3223	-0.40	0.20	-2.07	**0.03879**	-0.43	0.19	-2.25	**0.0241**	7%
374	RH_Default_Temp_8	Default	7.72	1.57	3.66	**0.0003**	0.00	0.00	-1.08	0.28072	-0.04	0.04	-0.98	0.3272	-0.40	0.20	-2.00	**0.04597**	-0.43	0.19	-2.20	**0.0282**	8%
439	Right_Cerebellum_IX	Cerebellum	8.22	1.82	3.70	**0.0002**	0.00	0.00	0.87	0.38372	0.03	0.04	0.96	0.3384	-0.47	0.20	-2.34	**0.01924**	-0.43	0.20	-2.21	**0.0269**	7%
94	LH_SalVentAttn_ParOper_3	Ventral Attention	7.32	1.65	3.75	**0.0002**	0.00	0.00	0.87	0.38470	0.02	0.03	0.95	0.3433	-0.46	0.20	-2.33	**0.01986**	-0.44	0.20	-2.27	**0.0231**	6%
2	LH_Vis_2	Visual	6.52	1.41	3.63	**0.0003**	0.00	0.00	0.87	0.38566	0.03	0.03	0.93	0.3512	-0.46	0.20	-2.32	**0.02054**	-0.43	0.19	-2.17	**0.0303**	6%
401	Left Thalamus	Subcortical	3.94	0.74	3.70	**0.0002**	-0.01	0.01	-0.99	0.32391	-0.03	0.03	-0.89	0.3713	-0.40	0.20	-2.06	**0.03971**	-0.44	0.20	-2.25	**0.0242**	8%
345	RH_Cont_PFCl_5	Frontoparietal	9.04	1.86	3.79	**0.0002**	0.00	0.00	-0.96	0.33691	-0.03	0.03	-0.87	0.3866	-0.40	0.20	-2.06	**0.03982**	-0.44	0.20	-2.20	**0.0275**	7%
304	RH_SalVentAttn_FrOper_3	Ventral Attention	5.27	1.12	3.74	**0.0002**	-0.01	0.01	-0.94	0.34828	-0.03	0.03	-0.86	0.3916	-0.41	0.20	-2.08	**0.03759**	-0.43	0.20	-2.21	**0.0268**	6%
400	RH_Default_PCC_9	Default	8.93	2.12	3.77	**0.0002**	0.00	0.00	0.77	0.44364	0.02	0.03	0.85	0.3933	-0.46	0.20	-2.33	**0.01998**	-0.43	0.20	-2.20	**0.0275**	5%
242	RH_SomMot_12	Somatomotor	7.90	1.55	3.58	**0.0003**	0.00	0.00	-0.92	0.35845	-0.03	0.03	-0.83	0.4048	-0.41	0.20	-2.06	**0.03927**	-0.44	0.20	-2.22	**0.0263**	7%
397	RH_Default_PCC_6	Default	9.59	1.88	3.78	**0.0002**	0.00	0.00	0.76	0.44966	0.03	0.04	0.83	0.4088	-0.46	0.19	-2.34	**0.01952**	-0.44	0.20	-2.23	**0.0257**	7%
342	RH_Cont_PFCl_2	Frontoparietal	9.02	1.97	3.71	**0.0002**	0.00	0.00	-0.86	0.38844	-0.03	0.03	-0.78	0.4334	-0.41	0.20	-2.14	**0.03203**	-0.44	0.20	-2.26	**0.0240**	6%
217	RH_Vis_17	Visual	6.37	1.50	3.74	**0.0002**	0.00	0.01	-0.90	0.37000	-0.03	0.04	-0.78	0.4348	-0.40	0.20	-2.12	**0.03387**	-0.44	0.20	-2.28	**0.0223**	7%
143	LH_Cont_PFCv_1	Frontoparietal	6.17	1.37	3.94	**0.0001**	0.00	0.00	-0.87	0.38407	-0.02	0.03	-0.78	0.4349	-0.41	0.20	-2.02	**0.04352**	-0.43	0.20	-2.20	**0.0276**	6%
41	LH_SomMot_10	Somatomotor	8.24	1.56	3.72	**0.0002**	0.00	0.00	0.72	0.47168	0.02	0.03	0.76	0.4451	-0.46	0.20	-2.26	**0.02407**	-0.43	0.20	-2.21	**0.0270**	5%
126	LH_Limbic_TempPole_8	Limbic	6.63	1.49	3.74	**0.0002**	0.00	0.00	-0.83	0.40729	-0.03	0.03	-0.73	0.4634	-0.41	0.20	-2.03	**0.04280**	-0.43	0.20	-2.22	**0.0264**	6%
293	RH_DorsAttn_PrCv_1	Dorsal Attention	7.92	1.55	3.70	**0.0002**	0.00	0.00	0.66	0.50849	0.02	0.03	0.71	0.4754	-0.46	0.20	-2.31	**0.02077**	-0.44	0.20	-2.26	**0.0239**	4%
152	LH_Default_Temp_4	Default	7.80	1.65	3.57	**0.0004**	0.00	0.00	0.62	0.53658	0.02	0.03	0.68	0.4978	-0.46	0.20	-2.29	**0.02195**	-0.44	0.20	-2.19	**0.0283**	5%
232	RH_SomMot_2	Somatomotor	7.97	1.55	3.75	**0.0002**	0.00	0.00	-0.72	0.47201	-0.03	0.04	-0.67	0.5002	-0.41	0.20	-2.16	**0.03105**	-0.44	0.20	-2.30	**0.0213**	6%
17	LH_Vis_17	Visual	10.37	2.23	3.86	**0.0001**	0.00	0.00	-0.71	0.47995	-0.02	0.03	-0.63	0.5257	-0.41	0.20	-2.08	**0.03763**	-0.44	0.20	-2.23	**0.0258**	5%
295	RH_SalVentAttn_TempOccPar_2	Ventral Attention	7.56	1.94	3.66	**0.0003**	0.00	0.00	-0.75	0.45407	-0.02	0.03	-0.62	0.5377	-0.42	0.20	-2.14	**0.03205**	-0.44	0.20	-2.24	**0.0250**	5%

240	RH_SomMot_10	Somatomotor	8.54	1.51	3.77	**0.0002**	0.00	0.00	-0.66	0.50928	-0.02	0.04	-0.61	0.5408	-0.41	0.20	-2.06	**0.03946**	-0.43	0.19	-2.20	**0.0277**	5%
71	LH_DorsAttn_Post_3	Dorsal Attention	11.05	2.16	3.71	**0.0002**	0.00	0.00	0.55	0.58037	0.02	0.04	0.61	0.5442	-0.45	0.20	-2.19	**0.02843**	-0.43	0.20	-2.12	**0.0340**	4%
33	LH_SomMot_2	Somatomotor	6.68	1.39	3.70	**0.0002**	0.00	0.00	0.56	0.57858	0.01	0.03	0.60	0.5473	-0.45	0.20	-2.27	**0.02293**	-0.43	0.20	-2.24	**0.0253**	3%
99	LH_SalVentAttn_FrOper_3	Ventral Attention	7.12	1.63	3.72	**0.0002**	0.00	0.00	-0.69	0.48998	-0.02	0.03	-0.59	0.5525	-0.41	0.20	-2.08	**0.03766**	-0.44	0.20	-2.21	**0.0272**	5%
245	RH_SomMot_15	Somatomotor	10.10	1.88	3.71	**0.0002**	0.00	0.00	0.54	0.59102	0.02	0.04	0.57	0.5661	-0.46	0.20	-2.28	**0.02271**	-0.44	0.20	-2.25	**0.0244**	4%
220	RH_Vis_20	Visual	9.14	1.56	3.68	**0.0002**	0.00	0.00	-0.59	0.55493	-0.03	0.04	-0.56	0.5728	-0.41	0.20	-2.06	**0.03930**	-0.43	0.20	-2.23	**0.0259**	6%
72	LH_DorsAttn_Post_4	Dorsal Attention	10.33	1.91	3.69	**0.0002**	0.00	0.00	-0.61	0.54474	-0.02	0.04	-0.56	0.5758	-0.41	0.20	-2.08	**0.03783**	-0.44	0.20	-2.25	**0.0242**	5%
246	RH_SomMot_16	Somatomotor	7.87	1.41	3.71	**0.0002**	0.00	0.00	0.52	0.60265	0.02	0.03	0.55	0.5822	-0.45	0.20	-2.32	**0.02027**	-0.43	0.19	-2.23	**0.0255**	4%
405	Left Hippocampus	Subcortical	5.88	1.15	3.64	**0.0003**	0.00	0.01	-0.58	0.56141	-0.02	0.03	-0.54	0.5908	-0.41	0.20	-2.02	**0.04356**	-0.43	0.20	-2.14	**0.0327**	4%
392	RH_Default_PCC_1	Default	7.45	1.47	3.77	**0.0002**	0.00	0.00	-0.59	0.55331	-0.02	0.04	-0.54	0.5924	-0.41	0.20	-2.14	**0.03207**	-0.44	0.20	-2.21	**0.0268**	5%
214	RH_Vis_14	Visual	7.54	1.42	3.70	**0.0002**	0.00	0.00	-0.57	0.56733	-0.02	0.04	-0.53	0.5954	-0.41	0.20	-2.08	**0.03764**	-0.43	0.20	-2.21	**0.0274**	5%
244	RH_SomMot_14	Somatomotor	7.35	1.58	3.73	**0.0002**	0.00	0.00	-0.58	0.56483	-0.02	0.03	-0.53	0.5957	-0.42	0.20	-2.06	**0.03972**	-0.43	0.20	-2.14	**0.0326**	4%
395	RH_Default_PCC_4	Default	6.91	1.56	3.75	**0.0002**	0.00	0.00	-0.58	0.55919	-0.02	0.03	-0.52	0.6051	-0.42	0.20	-2.17	**0.03027**	-0.43	0.20	-2.23	**0.0255**	4%
373	RH_Default_Temp_7	Default	7.45	1.64	3.74	**0.0002**	0.00	0.00	-0.58	0.56080	-0.02	0.03	-0.52	0.6063	-0.42	0.20	-2.06	**0.03924**	-0.43	0.20	-2.17	**0.0302**	4%
197	LH_Default_PCC_8	Default	7.88	1.75	3.64	**0.0003**	0.00	0.00	0.48	0.63450	0.01	0.03	0.51	0.6086	-0.45	0.20	-2.26	**0.02371**	-0.44	0.20	-2.18	**0.0295**	3%
22	LH_Vis_22	Visual	8.76	1.47	3.71	**0.0002**	0.00	0.00	-0.55	0.58482	-0.02	0.04	-0.51	0.6093	-0.41	0.20	-2.07	**0.03842**	-0.44	0.20	-2.19	**0.0286**	5%
238	RH_SomMot_8	Somatomotor	8.13	1.72	3.71	**0.0002**	0.00	0.00	-0.49	0.62332	-0.02	0.03	-0.44	0.6617	-0.42	0.20	-2.20	**0.02778**	-0.44	0.20	-2.30	**0.0216**	3%
36	LH_SomMot_5	Somatomotor	7.68	1.59	3.79	**0.0002**	0.00	0.00	-0.46	0.64716	-0.02	0.04	-0.42	0.6729	-0.42	0.20	-2.12	**0.03419**	-0.44	0.20	-2.26	**0.0238**	4%
20	LH_Vis_20	Visual	7.57	1.57	3.60	**0.0003**	0.00	0.00	0.38	0.70656	0.01	0.03	0.42	0.6738	-0.45	0.20	-2.30	**0.02157**	-0.43	0.20	-2.21	**0.0271**	3%
234	RH_SomMot_4	Somatomotor	6.49	1.28	3.69	**0.0002**	0.00	0.01	-0.44	0.66083	-0.01	0.04	-0.40	0.6908	-0.42	0.20	-2.09	**0.03705**	-0.43	0.20	-2.18	**0.0289**	3%
344	RH_Cont_PFCl_4	Frontoparietal	9.35	1.86	3.69	**0.0002**	0.00	0.00	-0.43	0.66358	-0.01	0.03	-0.40	0.6915	-0.42	0.20	-2.17	**0.03005**	-0.44	0.20	-2.24	**0.0248**	3%
398	RH_Default_PCC_7	Default	6.84	1.55	3.70	**0.0002**	0.00	0.00	0.32	0.74827	0.01	0.03	0.37	0.7094	-0.45	0.20	-2.24	**0.02515**	-0.43	0.20	-2.21	**0.0270**	2%
93	LH_SalVentAttn_ParOper_2	Ventral Attention	8.03	1.77	3.70	**0.0002**	0.00	0.00	-0.39	0.69366	-0.01	0.03	-0.37	0.7104	-0.42	0.20	-2.13	**0.03318**	-0.44	0.20	-2.24	**0.0250**	3%
248	RH_SomMot_18	Somatomotor	6.72	1.42	3.68	**0.0002**	0.00	0.00	0.34	0.73235	0.01	0.03	0.37	0.7106	-0.45	0.20	-2.19	**0.02846**	-0.44	0.20	-2.16	**0.0311**	2%
38	LH_SomMot_7	Somatomotor	8.35	1.67	3.69	**0.0002**	0.00	0.00	-0.34	0.73273	-0.01	0.03	-0.32	0.7478	-0.42	0.20	-2.12	**0.03441**	-0.44	0.20	-2.18	**0.0291**	3%
8	LH_Vis_8	Visual	11.80	2.02	3.71	**0.0002**	0.00	0.00	0.30	0.76288	0.01	0.04	0.32	0.7492	-0.45	0.20	-2.18	**0.02910**	-0.44	0.20	-2.20	**0.0276**	2%
40	LH_SomMot_9	Somatomotor	8.05	1.67	3.75	**0.0002**	0.00	0.00	0.28	0.78167	0.01	0.03	0.31	0.7557	-0.44	0.20	-2.26	**0.02388**	-0.43	0.20	-2.25	**0.0246**	2%
23	LH_Vis_23	Visual	8.44	2.02	3.73	**0.0002**	0.00	0.00	0.28	0.78149	0.01	0.03	0.31	0.7566	-0.45	0.20	-2.32	**0.02008**	-0.44	0.20	-2.30	**0.0216**	2%
37	LH_SomMot_6	Somatomotor	8.27	1.81	3.67	**0.0002**	0.00	0.00	-0.33	0.73790	-0.01	0.03	-0.30	0.7676	-0.42	0.20	-2.10	**0.03565**	-0.43	0.20	-2.18	**0.0293**	2%
224	RH_Vis_24	Visual	8.28	1.62	3.72	**0.0002**	0.00	0.00	-0.31	0.75767	-0.01	0.04	-0.26	0.7919	-0.42	0.19	-2.20	**0.02793**	-0.43	0.19	-2.25	**0.0245**	3%
307	RH_SalVentAttn_FrOper_6	Ventral Attention	6.65	1.36	3.71	**0.0002**	0.00	0.00	0.24	0.81173	0.01	0.03	0.25	0.8024	-0.44	0.20	-2.21	**0.02731**	-0.43	0.20	-2.19	**0.0286**	2%
441	Cerebellum_Vermis_X	Cerebellum	12.63	3.02	3.76	**0.0002**	0.00	0.00	0.22	0.82722	0.00	0.03	0.24	0.8085	-0.44	0.20	-2.27	**0.02315**	-0.43	0.20	-2.24	**0.0253**	1%
408	Right Thalamus	Subcortical	3.61	0.76	3.68	**0.0002**	0.00	0.01	0.21	0.83066	0.01	0.03	0.24	0.8104	-0.44	0.20	-2.22	**0.02645**	-0.44	0.20	-2.22	**0.0267**	2%
15	LH_Vis_15	Visual	7.84	1.95	3.73	**0.0002**	0.00	0.00	-0.23	0.81615	-0.01	0.03	-0.19	0.8464	-0.43	0.20	-2.22	**0.02632**	-0.44	0.20	-2.27	**0.0230**	2%
207	RH_Vis_7	Visual	6.66	1.51	3.68	**0.0002**	0.00	0.00	0.16	0.87432	0.01	0.03	0.18	0.8558	-0.44	0.20	-2.32	**0.02009**	-0.44	0.20	-2.32	**0.0202**	1%
6	LH_Vis_6	Visual	10.64	2.31	3.62	**0.0003**	0.00	0.00	-0.20	0.84240	0.00	0.03	-0.18	0.8560	-0.43	0.20	-2.09	**0.03642**	-0.43	0.20	-2.14	**0.0325**	1%
367	RH_Default_Temp_1	Default	7.53	1.50	3.72	**0.0002**	0.00	0.00	-0.17	0.86208	-0.01	0.04	-0.16	0.8704	-0.43	0.20	-2.18	**0.02942**	-0.44	0.20	-2.23	**0.0255**	2%
305	RH_SalVentAttn_FrOper_4	Ventral Attention	6.09	1.16	3.70	**0.0002**	0.00	0.01	0.13	0.89489	0.00	0.03	0.14	0.8870	-0.44	0.20	-2.24	**0.02535**	-0.44	0.19	-2.29	**0.0218**	1%
331	RH_Limbic_TempPole_7	Limbic	6.18	1.35	3.75	**0.0002**	0.00	0.01	0.11	0.91388	0.00	0.03	0.12	0.9031	-0.44	0.20	-2.23	**0.02593**	-0.43	0.19	-2.20	**0.0277**	1%
196	LH_Default_PCC_7	Default	9.27	1.99	3.69	**0.0002**	0.00	0.00	0.12	0.90709	0.00	0.03	0.12	0.9035	-0.44	0.20	-2.21	**0.02677**	-0.44	0.20	-2.25	**0.0243**	1%
70	LH_DorsAttn_Post_2	Dorsal Attention	8.74	1.68	3.78	**0.0002**	0.00	0.00	0.06	0.95202	0.00	0.04	0.07	0.9468	-0.44	0.20	-2.18	**0.02943**	-0.44	0.20	-2.20	**0.0276**	0%
159	LH_Default_Temp_11	Default	8.26	1.76	3.68	**0.0002**	0.00	0.00	0.05	0.95742	0.00	0.03	0.05	0.9562	-0.43	0.20	-2.19	**0.02837**	-0.43	0.19	-2.22	**0.0267**	0%
149	LH_Default_Temp_1	Default	7.14	1.58	3.82	**0.0001**	0.00	0.00	0.03	0.97321	0.00	0.03	0.03	0.9724	-0.43	0.20	-2.13	**0.03337**	-0.43	0.20	-2.13	**0.0328**	0%
16	LH_Vis_16	Visual	7.34	1.46	3.68	**0.0002**	0.00	0.00	-0.02	0.98060	0.00	0.03	-0.02	0.9846	-0.44	0.20	-2.29	**0.02216**	-0.44	0.20	-2.26	**0.0236**	0%

The mediation regression coefficients (ß), standard errors (SE), Z-scores and p-values for path a, b, ab, c and c'.

% mediation indicates the effect calculated from the proportion between path ab and path c

Supplementary Table 5. The potential brain age mediators of the relationships between CSVD and executive function (VFT)

ROI Index	Region	Network	*Path a*				*Path b*				*Paht ab*				*Path c'*				*Path c*				% mediation
			ß	SE	z	p	ß	SE	z	p	ß	SE	z	p	ß	SE	z	p	ß	SE	z	p	
392	RH_Default_PCC_1	Default	7.45	1.49	3.75	**0.0002**	-0.03	0.01	-2.17	**0.02963**	-0.20	0.10	-2.03	**0.0423**	-0.90	0.44	-2.07	**0.03867**	-1.09	0.42	-2.66	**0.0077**	18%
17	LH_Vis_17	Visual	10.40	2.21	3.78	**0.0002**	-0.02	0.01	-2.16	**0.03103**	-0.16	0.08	-2.02	**0.0436**	-0.93	0.43	-2.17	**0.02967**	-1.09	0.42	-2.65	**0.0081**	15%
244	RH_SomMot_14	Somatomotor	7.36	1.59	3.72	**0.0002**	-0.02	0.01	-1.97	**0.04841**	-0.15	0.08	-1.84	0.0656	-0.95	0.43	-2.28	**0.02232**	-1.10	0.42	-2.70	**0.0069**	13%
217	RH_Vis_17	Visual	6.35	1.51	3.77	**0.0002**	-0.02	0.01	-1.97	**0.04891**	-0.14	0.08	-1.82	0.0692	-0.95	0.43	-2.18	**0.02928**	-1.09	0.42	-2.62	**0.0088**	13%
202	RH_Vis_2	Visual	6.43	1.41	3.64	**0.0003**	-0.02	0.01	-1.96	0.05045	-0.14	0.08	-1.82	0.0693	-0.96	0.43	-2.21	**0.02732**	-1.10	0.42	-2.63	**0.0086**	13%
23	LH_Vis_23	Visual	8.46	2.03	3.70	**0.0002**	-0.02	0.01	-1.97	**0.04924**	-0.13	0.08	-1.78	0.0748	-0.97	0.42	-2.40	**0.01634**	-1.10	0.42	-2.70	**0.0069**	12%
149	LH_Default_Temp_1	Default	7.10	1.56	3.84	**0.0001**	-0.02	0.01	-1.85	0.06381	-0.16	0.09	-1.71	0.0876	-0.94	0.43	-2.27	**0.02308**	-1.10	0.42	-2.61	**0.0090**	14%
38	LH_SomMot_7	Somatomotor	8.36	1.66	3.70	**0.0002**	-0.02	0.01	-1.78	0.07470	-0.14	0.09	-1.69	0.0918	-0.95	0.43	-2.22	**0.02666**	-1.09	0.42	-2.55	**0.0107**	13%
429	Cerebellum_Vermis_VIIb	Cerebellum	8.69	1.99	3.83	**0.0001**	-0.01	0.01	-1.83	0.06780	-0.13	0.08	-1.66	0.0969	-0.97	0.42	-2.35	**0.01887**	-1.10	0.42	-2.71	**0.0068**	12%
405	Left Hippocampus	Subcortical	5.86	1.14	3.70	**0.0002**	-0.02	0.01	-1.75	0.08051	-0.14	0.09	-1.63	0.1039	-0.96	0.43	-2.20	**0.02793**	-1.10	0.42	-2.57	**0.0101**	12%
42	LH_SomMot_11	Somatomotor	8.06	1.67	3.82	**0.0001**	-0.02	0.01	-1.70	0.08852	-0.13	0.08	-1.58	0.1145	-0.96	0.43	-2.23	**0.02589**	-1.09	0.42	-2.56	**0.0106**	12%
331	RH_Limbic_TempPole_7	Limbic	6.19	1.37	3.74	**0.0002**	-0.02	0.01	-1.59	0.11094	-0.12	0.08	-1.51	0.1314	-0.96	0.43	-2.20	**0.02808**	-1.09	0.42	-2.55	**0.0107**	11%
395	RH_Default_PCC_4	Default	6.90	1.53	3.75	**0.0002**	-0.02	0.01	-1.56	0.11847	-0.11	0.08	-1.43	0.1520	-0.98	0.43	-2.29	**0.02212**	-1.10	0.42	-2.68	**0.0074**	10%
36	LH_SomMot_5	Somatomotor	7.67	1.58	3.78	**0.0002**	-0.02	0.01	-1.47	0.14194	-0.12	0.09	-1.37	0.1722	-0.98	0.43	-2.26	**0.02399**	-1.10	0.42	-2.57	**0.0102**	11%
306	RH_SalVentAttn_FrOper_5	Ventral Attention	8.51	1.38	3.66	**0.0003**	-0.02	0.01	-1.41	0.15818	-0.15	0.12	-1.36	0.1744	-0.94	0.45	-2.12	**0.03374**	-1.10	0.42	-2.60	**0.0092**	14%
152	LH_Default_Temp_4	Default	7.78	1.65	3.61	**0.0003**	-0.02	0.01	-1.39	0.16573	-0.12	0.09	-1.30	0.1929	-0.97	0.43	-2.32	**0.02046**	-1.09	0.42	-2.69	**0.0071**	11%
242	RH_SomMot_12	Somatomotor	7.89	1.54	3.60	**0.0003**	-0.01	0.01	-1.39	0.16378	-0.11	0.09	-1.30	0.1941	-0.99	0.42	-2.42	**0.01540**	-1.10	0.42	-2.74	**0.0062**	10%
307	RH_SalVentAttn_FrOper_6	Ventral Attention	6.59	1.38	3.79	**0.0002**	-0.02	0.01	-1.39	0.16599	-0.11	0.09	-1.25	0.2115	-0.98	0.43	-2.27	**0.02301**	-1.09	0.42	-2.64	**0.0083**	10%
37	LH_SomMot_6	Somatomotor	8.24	1.82	3.68	**0.0002**	-0.01	0.01	-1.34	0.17959	-0.09	0.08	-1.24	0.2161	-1.01	0.43	-2.40	**0.01645**	-1.10	0.42	-2.76	**0.0059**	9%
245	RH_SomMot_15	Somatomotor	10.07	1.85	3.75	**0.0002**	-0.01	0.01	-1.32	0.18764	-0.11	0.09	-1.23	0.2178	-0.98	0.43	-2.25	**0.02444**	-1.10	0.42	-2.50	**0.0125**	10%
41	LH_SomMot_10	Somatomotor	8.24	1.58	3.72	**0.0002**	-0.01	0.01	-1.30	0.19491	-0.11	0.09	-1.22	0.2241	-0.99	0.43	-2.37	**0.01797**	-1.10	0.42	-2.69	**0.0072**	10%
40	LH_SomMot_9	Somatomotor	8.05	1.66	3.74	**0.0002**	-0.01	0.01	-1.28	0.20090	-0.10	0.08	-1.21	0.2257	-1.00	0.43	-2.38	**0.01727**	-1.10	0.42	-2.66	**0.0078**	9%
214	RH_Vis_14	Visual	7.55	1.40	3.70	**0.0002**	-0.01	0.01	-1.30	0.19384	-0.11	0.09	-1.21	0.2280	-0.99	0.44	-2.39	**0.01702**	-1.10	0.42	-2.71	**0.0066**	10%
224	RH_Vis_24	Visual	8.27	1.61	3.77	**0.0002**	-0.01	0.01	-1.29	0.19791	-0.10	0.09	-1.19	0.2357	-0.98	0.43	-2.27	**0.02295**	-1.09	0.42	-2.66	**0.0078**	9%
126	LH_Limbic_TempPole_8	Limbic	6.65	1.49	3.69	**0.0002**	-0.02	0.01	-1.28	0.20059	-0.10	0.09	-1.17	0.2402	-0.99	0.43	-2.31	**0.02112**	-1.09	0.42	-2.67	**0.0076**	9%
401	Left Thalamus	Subcortical	3.93	0.72	3.75	**0.0002**	-0.03	0.02	-1.23	0.21900	-0.10	0.08	-1.14	0.2531	-1.00	0.43	-2.38	**0.01737**	-1.10	0.42	-2.71	**0.0066**	9%
121	LH_Limbic_TempPole_3	Limbic	6.74	1.69	3.70	**0.0002**	-0.01	0.01	-1.29	0.19785	-0.09	0.08	-1.13	0.2595	-1.00	0.43	-2.36	**0.01840**	-1.10	0.42	-2.66	**0.0078**	8%
240	RH_SomMot_10	Somatomotor	8.56	1.50	3.72	**0.0002**	-0.01	0.01	-1.10	0.27310	-0.10	0.09	-1.03	0.3019	-0.99	0.43	-2.30	**0.02128**	-1.10	0.42	-2.67	**0.0076**	9%
93	LH_SalVentAttn_ParOper_2	Ventral Attention	8.08	1.78	3.66	**0.0003**	-0.01	0.01	-1.03	0.30475	-0.07	0.07	-0.95	0.3429	-1.03	0.43	-2.43	**0.01530**	-1.10	0.42	-2.58	**0.0100**	7%
351	RH_Cont_PFCl_11	Frontoparietal	8.65	1.81	3.64	**0.0003**	0.01	0.01	0.89	0.37608	0.06	0.08	0.95	0.3439	-1.16	0.43	-2.71	**0.00679**	-1.10	0.42	-2.70	**0.0069**	6%
231	RH_SomMot_1	Somatomotor	5.98	1.18	3.72	**0.0002**	-0.01	0.01	-1.02	0.30739	-0.08	0.08	-0.93	0.3538	-1.02	0.43	-2.41	**0.01576**	-1.10	0.42	-2.64	**0.0084**	7%
408	Right Thalamus	Subcortical	3.61	0.76	3.68	**0.0002**	-0.02	0.02	-1.00	0.31719	-0.08	0.08	-0.91	0.3644	-1.02	0.43	-2.38	**0.01722**	-1.10	0.42	-2.59	**0.0097**	7%
304	RH_SalVentAttn_FrOper_3	Ventral Attention	5.28	1.12	3.74	**0.0002**	-0.01	0.01	-0.97	0.33330	-0.07	0.08	-0.89	0.3739	-1.03	0.43	-2.44	**0.01483**	-1.09	0.42	-2.64	**0.0083**	6%
71	LH_DorsAttn_Post_3	Dorsal Attention	11.03	2.14	3.74	**0.0002**	-0.01	0.01	-0.91	0.36089	-0.08	0.09	-0.83	0.4066	-1.02	0.44	-2.30	**0.02159**	-1.10	0.42	-2.56	**0.0106**	7%
398	RH_Default_PCC_7	Default	6.81	1.55	3.77	**0.0002**	-0.01	0.01	-0.89	0.37556	-0.06	0.07	-0.78	0.4349	-1.04	0.42	-2.48	**0.01329**	-1.10	0.42	-2.70	**0.0069**	5%
374	RH_Default_Temp_8	Default	7.69	1.57	3.70	**0.0002**	-0.01	0.01	-0.86	0.38826	-0.07	0.09	-0.78	0.4364	-1.02	0.43	-2.43	**0.01510**	-1.09	0.42	-2.57	**0.0101**	7%
342	RH_Cont_PFCl_2	Frontoparietal	9.04	1.96	3.68	**0.0002**	-0.01	0.01	-0.84	0.40319	-0.07	0.08	-0.76	0.4459	-1.03	0.43	-2.39	**0.01691**	-1.09	0.42	-2.59	**0.0096**	6%
15	LH_Vis_15	Visual	7.81	1.97	3.77	**0.0002**	0.01	0.01	0.64	0.52438	0.05	0.07	0.75	0.4555	-1.14	0.42	-2.66	**0.00773**	-1.09	0.42	-2.64	**0.0082**	4%
400	RH_Default_PCC_9	Default	8.89	2.12	3.76	**0.0002**	0.00	0.01	0.65	0.51598	0.04	0.07	0.74	0.4576	-1.14	0.43	-2.69	**0.00711**	-1.10	0.42	-2.62	**0.0087**	4%
441	Cerebellum_Vermis_X	Cerebellum	12.66	3.00	3.75	**0.0002**	0.00	0.01	-0.74	0.45783	-0.06	0.08	-0.65	0.5152	-1.04	0.44	-2.37	**0.01793**	-1.10	0.43	-2.53	**0.0114**	5%
155	LH_Default_Temp_7	Default	7.79	1.67	3.72	**0.0002**	0.01	0.01	0.60	0.55077	0.04	0.08	0.65	0.5167	-1.14	0.43	-2.58	**0.00987**	-1.09	0.42	-2.59	**0.0095**	4%
207	RH_Vis_7	Visual	6.64	1.49	3.71	**0.0002**	-0.01	0.01	-0.71	0.47628	-0.05	0.07	-0.65	0.5167	-1.05	0.43	-2.38	**0.01731**	-1.10	0.42	-2.53	**0.0114**	4%
234	RH_SomMot_4	Somatomotor	6.49	1.26	3.68	**0.0002**	0.01	0.01	0.59	0.55590	0.05	0.09	0.65	0.5172	-1.14	0.44	-2.64	**0.00840**	-1.09	0.42	-2.62	**0.0087**	5%
345	RH_Cont_PFCl_5	Frontoparietal	9.02	1.84	3.79	**0.0002**	-0.01	0.01	-0.70	0.48347	-0.05	0.08	-0.63	0.5289	-1.04	0.43	-2.45	**0.01432**	-1.09	0.42	-2.62	**0.0088**	5%
197	LH_Default_PCC_8	Default	7.84	1.75	3.72	**0.0002**	-0.01	0.01	-0.70	0.48611	-0.05	0.08	-0.62	0.5324	-1.04	0.42	-2.49	**0.01283**	-1.09	0.41	-2.65	**0.0080**	5%

198	LH_Default_PCC_9	Default	7.27	1.61	3.67	**0.0002**	-0.01	0.01	-0.64	0.52169	-0.04	0.07	-0.59	0.5584	-1.06	0.43	-2.51	**0.01193**	-1.10	0.42	-2.67	**0.0075**	4%
246	RH_SomMot_16	Somatomotor	7.86	1.44	3.69	**0.0002**	-0.01	0.01	-0.62	0.53545	-0.05	0.08	-0.58	0.5603	-1.04	0.43	-2.46	**0.01397**	-1.09	0.42	-2.60	**0.0094**	5%
248	RH_SomMot_18	Somatomotor	6.71	1.41	3.69	**0.0002**	-0.01	0.01	-0.63	0.52803	-0.04	0.07	-0.58	0.5611	-1.05	0.43	-2.47	**0.01339**	-1.09	0.42	-2.63	**0.0086**	4%
44	LH_SomMot_13	Somatomotor	8.75	1.51	3.66	**0.0003**	0.01	0.01	0.55	0.58108	0.05	0.09	0.58	0.5633	-1.14	0.43	-2.76	**0.00582**	-1.10	0.42	-2.65	**0.0081**	4%
305	RH_SalVentAttn_FrOper_4	Ventral Attention	6.08	1.16	3.73	**0.0002**	-0.01	0.01	-0.61	0.54060	-0.05	0.09	-0.57	0.5690	-1.04	0.43	-2.38	**0.01732**	-1.09	0.42	-2.56	**0.0104**	5%
43	LH_SomMot_12	Somatomotor	7.36	1.53	3.74	**0.0002**	-0.01	0.01	-0.60	0.54612	-0.04	0.08	-0.55	0.5800	-1.05	0.43	-2.51	**0.01203**	-1.09	0.42	-2.61	**0.0091**	4%
434	Left_Cerebellum_VIIIb	Cerebellum	7.05	1.59	3.61	**0.0003**	-0.01	0.01	-0.63	0.52869	-0.05	0.08	-0.54	0.5912	-1.05	0.43	-2.44	**0.01479**	-1.10	0.41	-2.62	**0.0088**	5%
35	LH_SomMot_4	Somatomotor	6.32	1.37	3.71	**0.0002**	-0.01	0.01	-0.59	0.55716	-0.05	0.08	-0.53	0.5929	-1.04	0.43	-2.42	**0.01570**	-1.09	0.42	-2.67	**0.0076**	4%
350	RH_Cont_PFCl_10	Frontoparietal	7.44	1.61	3.77	**0.0002**	-0.01	0.01	-0.60	0.54875	-0.04	0.07	-0.53	0.5931	-1.05	0.43	-2.55	**0.01067**	-1.10	0.42	-2.69	**0.0072**	4%
20	LH_Vis_20	Visual	7.53	1.58	3.62	**0.0003**	-0.01	0.01	-0.57	0.57191	-0.04	0.09	-0.53	0.5993	-1.05	0.43	-2.48	**0.01324**	-1.09	0.41	-2.66	**0.0077**	4%
8	LH_Vis_8	Visual	11.81	1.96	3.73	**0.0002**	0.00	0.01	-0.56	0.57260	-0.05	0.10	-0.52	0.6037	-1.05	0.42	-2.57	**0.01025**	-1.10	0.41	-2.76	**0.0058**	4%
220	RH_Vis_20	Visual	9.10	1.53	3.72	**0.0002**	0.00	0.01	0.45	0.65568	0.04	0.10	0.48	0.6335	-1.13	0.44	-2.49	**0.01265**	-1.09	0.42	-2.50	**0.0125**	4%
45	LH_SomMot_14	Somatomotor	6.72	1.56	3.77	**0.0002**	0.00	0.01	-0.53	0.59958	-0.03	0.07	-0.45	0.6532	-1.06	0.43	-2.56	**0.01039**	-1.10	0.42	-2.69	**0.0072**	3%
70	LH_DorsAttn_Post_2	Dorsal Attention	8.73	1.66	3.74	**0.0002**	0.00	0.01	0.39	0.70022	0.03	0.09	0.41	0.6840	-1.13	0.43	-2.55	**0.01085**	-1.09	0.42	-2.54	**0.0112**	3%
397	RH_Default_PCC_6	Default	9.63	1.90	3.76	**0.0002**	0.00	0.01	-0.45	0.65130	-0.04	0.08	-0.41	0.6841	-1.06	0.43	-2.50	**0.01254**	-1.10	0.42	-2.62	**0.0088**	3%
295	RH_SalVentAttn_TempOccPar_2	Ventral Attention	7.57	1.95	3.64	**0.0003**	0.00	0.01	0.33	0.73947	0.02	0.08	0.39	0.6940	-1.12	0.42	-2.65	**0.00796**	-1.09	0.42	-2.61	**0.0090**	2%
102	LH_SalVentAttn_FrOper_6	Ventral Attention	7.10	1.55	3.68	**0.0002**	0.00	0.01	-0.42	0.67191	-0.03	0.08	-0.39	0.6988	-1.05	0.43	-2.40	**0.01653**	-1.09	0.42	-2.57	**0.0103**	3%
141	LH_Cont_PFCl_8	Frontoparietal	7.51	1.56	3.68	**0.0002**	0.00	0.01	0.34	0.73654	0.02	0.07	0.37	0.7147	-1.12	0.43	-2.63	**0.00863**	-1.09	0.42	-2.60	**0.0093**	2%
367	RH_Default_Temp_1	Default	7.50	1.47	3.73	**0.0002**	-0.01	0.01	-0.40	0.69240	-0.04	0.10	-0.36	0.7163	-1.06	0.44	-2.51	**0.01205**	-1.10	0.42	-2.63	**0.0085**	4%
26	LH_Vis_26	Visual	7.36	1.58	3.78	**0.0002**	0.00	0.01	-0.38	0.70372	-0.03	0.08	-0.34	0.7360	-1.07	0.43	-2.50	**0.01232**	-1.10	0.42	-2.57	**0.0101**	2%
232	RH_SomMot_2	Somatomotor	7.97	1.53	3.73	**0.0002**	0.00	0.01	-0.36	0.72137	-0.03	0.09	-0.32	0.7524	-1.06	0.42	-2.47	**0.01350**	-1.09	0.41	-2.61	**0.0091**	3%
191	LH_Default_PCC_2	Default	6.98	1.59	3.73	**0.0002**	0.00	0.01	-0.33	0.74394	-0.03	0.08	-0.28	0.7760	-1.07	0.43	-2.51	**0.01223**	-1.09	0.42	-2.60	**0.0093**	3%
439	Right_Cerebellum_IX	Cerebellum	8.22	1.81	3.66	**0.0003**	0.00	0.01	-0.31	0.75478	-0.02	0.08	-0.28	0.7774	-1.07	0.43	-2.45	**0.01421**	-1.09	0.42	-2.56	**0.0103**	2%
236	RH_SomMot_6	Somatomotor	8.93	1.53	3.68	**0.0002**	0.00	0.01	0.27	0.78914	0.03	0.10	0.28	0.7776	-1.14	0.44	-2.64	**0.00829**	-1.11	0.42	-2.74	**0.0061**	3%
2	LH_Vis_2	Visual	6.50	1.41	3.64	**0.0003**	0.00	0.01	-0.30	0.76545	-0.02	0.08	-0.27	0.7854	-1.08	0.43	-2.50	**0.01226**	-1.10	0.42	-2.61	**0.0090**	2%
293	RH_DorsAttn_PrCv_1	Dorsal Attention	7.95	1.53	3.66	**0.0002**	0.00	0.01	-0.28	0.78314	-0.02	0.07	-0.25	0.8001	-1.08	0.43	-2.52	**0.01179**	-1.10	0.42	-2.67	**0.0075**	2%
159	LH_Default_Temp_11	Default	8.26	1.76	3.69	**0.0002**	0.00	0.01	-0.28	0.78139	-0.02	0.07	-0.25	0.8007	-1.08	0.43	-2.54	**0.01118**	-1.10	0.42	-2.60	**0.0092**	2%
107	LH_SalVentAttn_Med_1	Ventral Attention	6.79	1.28	3.67	**0.0002**	0.00	0.01	0.21	0.83720	0.01	0.08	0.22	0.8294	-1.11	0.43	-2.68	**0.00741**	-1.10	0.42	-2.66	**0.0078**	1%
99	LH_SalVentAttn_FrOper_3	Ventral Attention	7.12	1.62	3.72	**0.0002**	0.00	0.01	-0.23	0.81486	-0.02	0.07	-0.21	0.8367	-1.07	0.44	-2.48	**0.01302**	-1.09	0.42	-2.55	**0.0108**	2%
22	LH_Vis_22	Visual	8.76	1.46	3.72	**0.0002**	0.00	0.01	0.18	0.85728	0.02	0.10	0.20	0.8443	-1.11	0.43	-2.67	**0.00762**	-1.10	0.41	-2.67	**0.0075**	1%
196	LH_Default_PCC_7	Default	9.31	2.02	3.65	**0.0003**	0.00	0.01	0.18	0.85883	0.01	0.08	0.19	0.8518	-1.12	0.43	-2.72	**0.00659**	-1.10	0.41	-2.77	**0.0056**	1%
6	LH_Vis_6	Visual	10.59	2.31	3.69	**0.0002**	0.00	0.01	-0.19	0.84877	-0.02	0.08	-0.17	0.8685	-1.08	0.43	-2.51	**0.01198**	-1.10	0.42	-2.71	**0.0067**	1%
160	LH_Default_Temp_12	Default	10.06	1.99	3.66	**0.0003**	0.00	0.01	0.15	0.88423	0.01	0.09	0.16	0.8712	-1.11	0.43	-2.61	**0.00906**	-1.10	0.42	-2.61	**0.0089**	1%
235	RH_SomMot_5	Somatomotor	6.54	1.49	4.17	**0.0000**	0.00	0.01	-0.20	0.84452	-0.02	0.08	-0.16	0.8717	-1.07	0.43	-2.50	**0.01232**	-1.09	0.42	-2.54	**0.0110**	2%
94	LH_SalVentAttn_ParOper_3	Ventral Attention	7.37	1.66	3.70	**0.0002**	0.00	0.01	-0.19	0.85112	-0.01	0.07	-0.16	0.8722	-1.08	0.42	-2.58	**0.00988**	-1.10	0.42	-2.62	**0.0089**	1%
16	LH_Vis_16	Visual	7.35	1.45	3.68	**0.0002**	0.00	0.01	-0.17	0.86400	-0.01	0.09	-0.15	0.8778	-1.09	0.43	-2.62	**0.00870**	-1.11	0.42	-2.68	**0.0073**	1%
48	LH_SomMot_17	Somatomotor	7.03	1.41	3.60	**0.0003**	0.00	0.01	0.12	0.90519	0.01	0.08	0.14	0.8925	-1.11	0.42	-2.65	**0.00795**	-1.10	0.41	-2.76	**0.0057**	1%
311	RH_SalVentAttn_Med_1	Ventral Attention	6.37	1.29	3.65	**0.0003**	0.00	0.01	0.12	0.90578	0.01	0.07	0.12	0.9022	-1.10	0.43	-2.62	**0.00879**	-1.10	0.42	-2.66	**0.0078**	1%
373	RH_Default_Temp_7	Default	7.50	1.65	3.67	**0.0002**	0.00	0.01	0.09	0.92547	0.00	0.08	0.10	0.9183	-1.10	0.43	-2.65	**0.00801**	-1.10	0.42	-2.64	**0.0082**	0%
72	LH_DorsAttn_Post_4	Dorsal Attention	10.31	1.89	3.70	**0.0002**	0.00	0.01	-0.09	0.92550	-0.01	0.09	-0.09	0.9267	-1.09	0.43	-2.61	**0.00915**	-1.10	0.42	-2.72	**0.0065**	1%
33	LH_SomMot_2	Somatomotor	6.68	1.39	3.70	**0.0002**	0.00	0.01	-0.08	0.93648	-0.01	0.07	-0.07	0.9453	-1.09	0.44	-2.50	**0.01257**	-1.09	0.42	-2.63	**0.0085**	1%
344	RH_Cont_PFCl_4	Frontoparietal	9.36	1.84	3.69	**0.0002**	0.00	0.01	-0.07	0.94379	-0.01	0.08	-0.07	0.9474	-1.09	0.43	-2.54	**0.01121**	-1.09	0.42	-2.63	**0.0086**	1%
238	RH_SomMot_8	Somatomotor	8.18	1.77	3.64	**0.0003**	0.00	0.01	-0.07	0.94482	0.00	0.08	-0.06	0.9484	-1.09	0.43	-2.60	**0.00924**	-1.09	0.42	-2.67	**0.0076**	0%
143	LH_Cont_PFCv_1	Frontoparietal	6.14	1.39	4.01	**0.0001**	0.00	0.01	0.03	0.97937	0.00	0.07	0.03	0.9722	-1.10	0.43	-2.54	**0.01121**	-1.10	0.42	-2.60	**0.0092**	0%

The mediation regression coefficients (ß), standard errors (SE), Z-scores and p-values for path a, b, ab, c and c'.

% mediation indicates the effect calculated from the proportion between path ab and path c

Supplementary Figure 1. The histogram of chronological age in each study site.

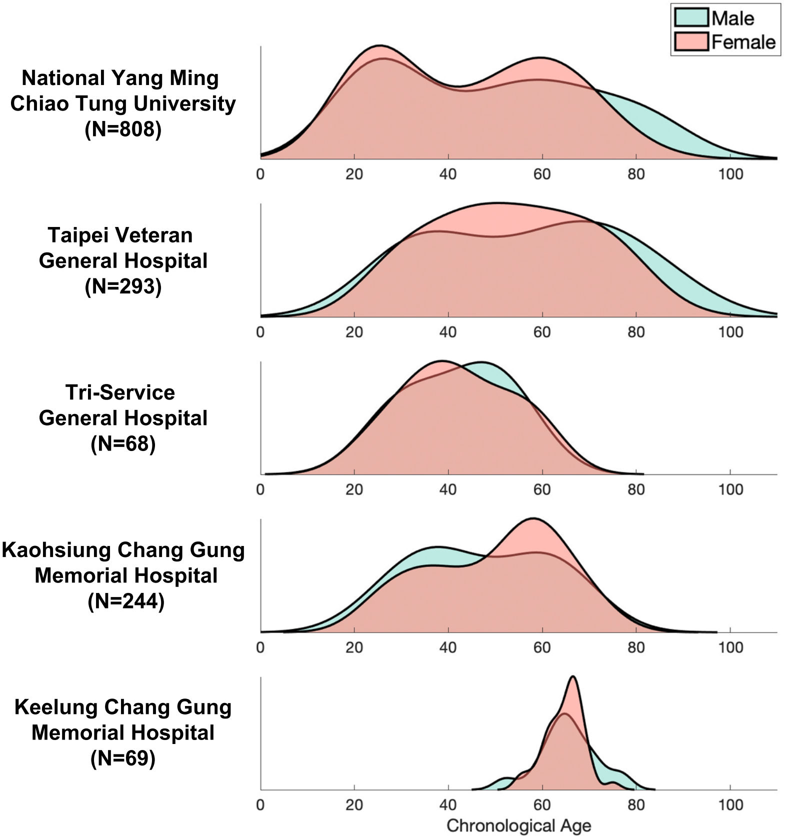

Supplementary Figure 2. The overview of data recruitment.

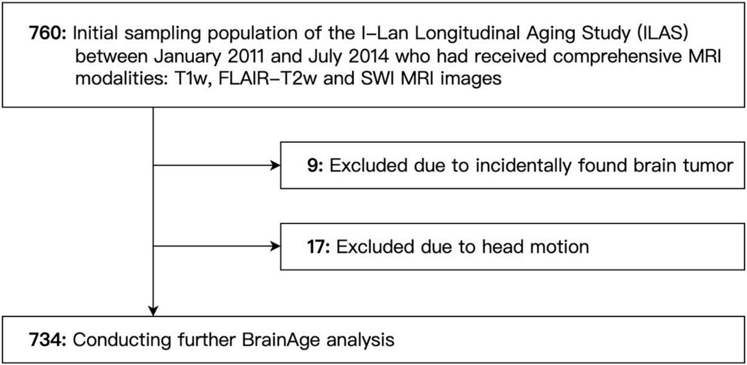

T1w=T1 weighted image; FLAIR=fluid-attenuated inversion recovery; SWI=susceptibility-weighted images.

Supplementary Figure 3. Histogram of white matter hyperintensity volumes, lacune and cerebral microbleeds in the ILAS population

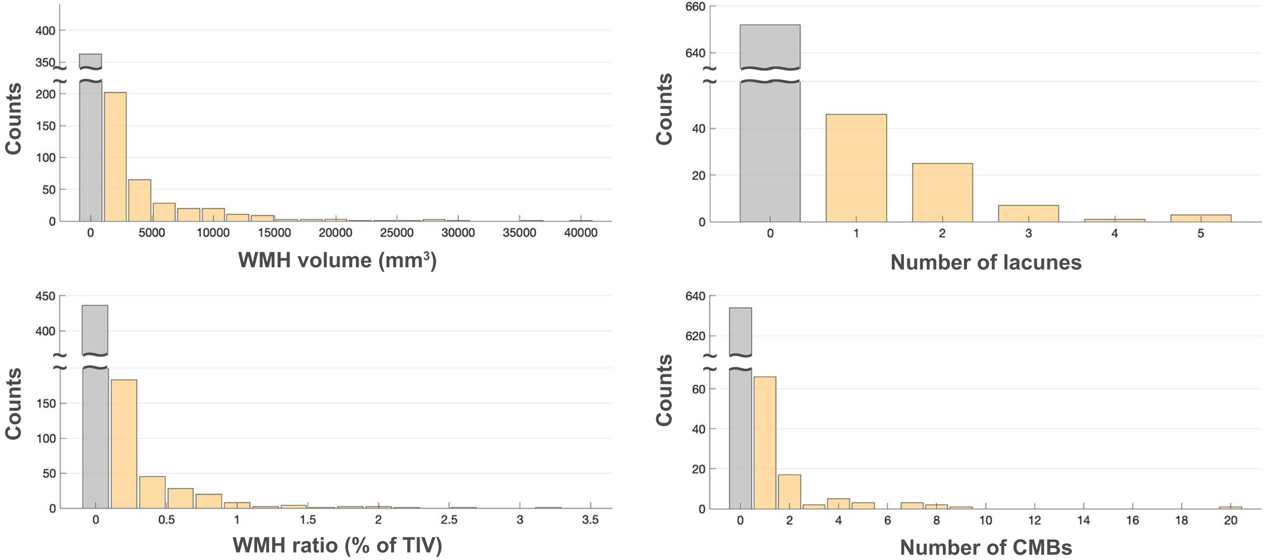

Supplement: fcac233_Supplementary_Data [file fcac233_supplementary_data.pdf]
